# Electrochemical and quantum chemical studies on the corrosion inhibition of 1037 carbon steel by different types of surfactants

**DOI:** 10.1039/d1ra07983b

**Published:** 2022-01-24

**Authors:** Abd El-Aziz S. Fouda, Ameena M. Al-bonayan, Mohamed Eissa, Dalia M. Eid

**Affiliations:** Chemistry Department, Faculty of Science, Mansoura University Mansoura-35516 Egypt asfouda@hotmail.com asfouda@mans.edu.eg +2 050 2202264 +2 050 2365730; Chemistry Department, Faculty of Science, Umm Al-Qura University Makkah Saudi Arabia benayana@hotmail.com; Higher Institute of Engineering & Technology, Kma Alex Egypt; College of Science &Humanities-Harimlae, Shaqra University Kingdom of Saudi Arabia; Chemistry and Earth Science Department, Qatar University Doha-2713 Qatar

## Abstract

In this work, three different types of surfactants, namely, dodecyl trimethyl ammonium chloride (DTAC, C_12_H_25_N (CH_3_)_3_Cl)^−^, octyl phenol poly(ethylene glycol ether)_*x*_ (TX-100, C_34_H_62_O_11_ for *x* = 10) and dioctyl sodium sulfosuccinate (AOT-100, C_20_H_37_O_7_NaS) with corrosion restraint were utilized as corrosion inhibitors for 1037 CS in 0.5 M HCl. The protection efficacy (% IE) was indicated by weight loss and electrochemical measurements. Polarization curves showed that the investigated compounds are mixed-type inhibitors. The protection efficacy (% IE) increases with the increase in the surfactant concentration and reached 64.42–86.46% at 8 × 10^−4^ M and 30 °C. Adsorption of these utilized surfactants (DTAC, TX-100, and AOT) onto the CS surface concurred with the Langmuir adsorption isotherm. Impedance data revealed that by increasing the surfactant concentration, the charge transfer resistance (*R*_ct_) increases and *vice versa* for the capacitance of double layer (*C*_dl_). Surface morphological investigations such as scanning electron microscopy (SEM) combined with EDX and atomic force microscopy (AFM) were used to further investigate the inhibitors' protective abilities. Monte Carlo simulations showed the great interaction between the tested surfactants and the metal surface of the CS. The theoretical results (density functional theory, DFT) were in good agreement with experimental measurements. The restraint efficiencies of anionic, neutral, and cationic surfactants regarded a certain dating to HSAB precept and Fukui indices.

## Abstract 2

In this work, three different types of surfactants DTAC, C_12_H_25_N (CH_3_)_3_Cl^−^, TX-100, C_34_H_62_O_11_ for *x* = 10) and AOT-100, C_20_H_37_O_7_NaS) with corrosion restraint were utilized as corrosion inhibitors for 1037 CS in 0.5 M HCl.

## Graphical abstract

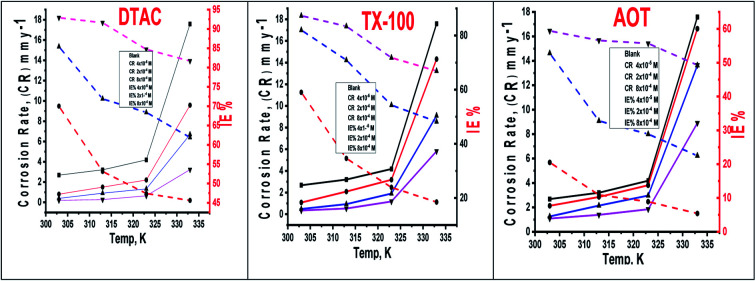

## 1. Introduction

The hydrochloric acid solution was utilized as a pickling agent in oil fields because it is the most inexpensive manner to interrupt down calcium carbonate, CaCO_3_, scale interior the pipelines beneath maximum conditions. Appropriately, corrosion surfactants (regularly surfactants) should be injected with the hydrochloric acid solution to avoid the adverse impact of acid on the surface of the pipelines.^1^ CS has been extensively applied as a production material for pipe paintings inside the oil and fuel lines, which include down hollow tubular, float lines, and transmission pipelines.^2^ Surfactants are compounds that may be determined in a huge variety of business settings to analyze laboratories and are part of our everyday lives. The surfactants have severe focal factors including excessive inhibition efficiency, low price, low toxicity, and clean manufacturing.^3^ Moreover, research on surfactants adsorbed onto steel surfaces is extraordinarily critical for electrochemical properties such as corrosion inhibition, adhesion, lubrication, and detergency.^4^ “The effective type of corrosion surfactant for these applications is film-forming surfactant. Nowadays, surfactants are widely used, and find a wide range of applications in the petroleum industry. This is attributed to their remarkable ability to influence the properties of surfaces and interfaces. Recently, one of the most important applications of surfactants is effective corrosion inhibition in the oil industry. It interacts with anodic or cathodic reaction sites and retards corrosive oxidation and reduction reactions. An increment in surfactant action was observed when the concentration within the destructive arrangement approaches the basic critical micelle concentration (CMC). Above this value, there was no further increment in the efficiency that remains constant for further increment in surfactant concentration. Within the nonappearance of a charged head group, the driving force of micellization is the hydrophobic drive and van der Waals attractions. The strong interaction between water particles repulses the hydrocarbon chain from the water bulk phase. This drives the surfactants to form aggregates where the hydrophilic head groups conceal the hydrocarbon chains. It was observed that the adsorption of these surfactants depends on the physico-chemical properties of the useful groups and the electron density at the donor atoms. The adsorption happens due to the interaction of the lone pair and/or π-orbitals of surfactants with the d-orbitals of the metal surface atoms, which brings out more prominent adsorption of the surfactant particles onto the surface, leading to the formation of a corrosion protection film.^5–7^ The adsorption is additionally affected by the structure and the charge of the metal surface, and the type of testing electrolyte.^8–10^ Recently, quantum chemical strategies have been demonstrated to be exceptionally valuable in deciding the atomic structure as well as elucidating the electronic structure and reactivity.^11^ In this way, it has gotten to be a common practice to carry out quantum chemical calculations in corrosion hindrance studies. The concept of evaluating the productivity of a corrosion surfactant by computational chemistry is to search for compounds with desirable properties using chemical intuition and experience into a mathematically quantified and computerized form. Once a relationship between the structure and movement or property is found, any number of compounds, counting those not however synthesized, can be promptly screened by a computational technique^12^ and a set of numerical conditions, which can speak to the chemical phenomenon beneath study.^13,14^ Surfactants, because of their remarkable ability to influence the properties of surfaces and interfaces, have been effectively exploited as corrosion surfactants in an acidic medium.^15–25^ Surfactants are amphiphilic molecules containing one hydrophilic (head) and other hydrophobic (tail) parts; this favors the adsorption process at metallic surfaces”. The hydrophilic part of the surfactant can be positive, negative, impartial or zwitterionic and the hydrophobic component includes one or more hydrocarbon chains, commonly with 6–22 carbon atoms.

The aim of this paper is to get data on the level of corrosion activity in a system, utilizing the chemical and electrochemical procedures. This information is utilized to assess the corrosion surfactant capability. Another objective of this work is to calculate the more related molecular properties based on its activity as corrosion inhibitors. The nearby reactivity was analyzed using Fukui indices, since they indicate the reactive regions within the shape of the nucleophilic and electrophilic behavior of each atom in the molecule. These surfactants are not used as corrosion inhibitors for CS in HCl media in the literature.

## 2. Experimental

### 2.1 Chemicals and materials

**Scheme 1:**
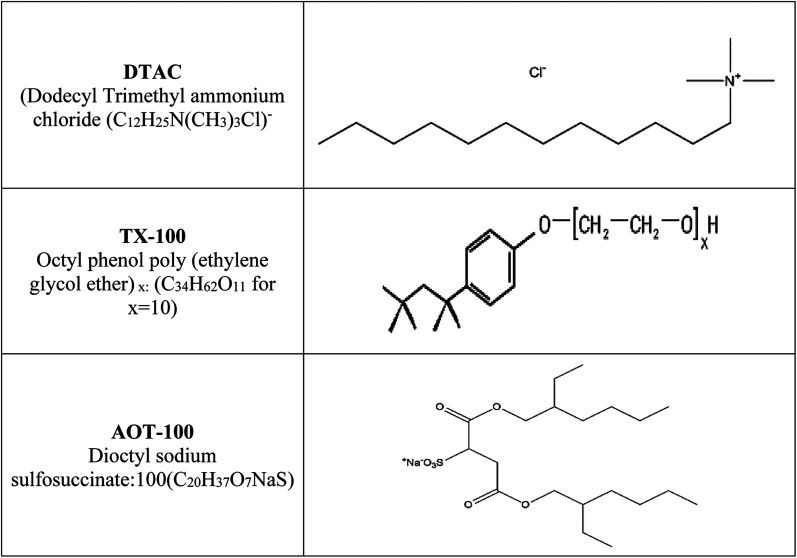
The structure, names and molecular formulas of the utilized surfactants.

Hydrochloric acid (37% wt), “ethyl alcohol and acetone were acquired from Al-Gomhoria Company (Egypt). Surfactants (dodecyl trimethyl ammonium chloride: DTAC (C_12_H_25_N (CH_3_)_3_ –Cl); octyl phenol poly(ethylene glycol ether)_*x*_: TX-100 (C_34_H_62_O_11_ for *x* = 10); dioctyl sodium sulfosuccinate: AOT-100 (C_20_H_37_O_7_NaS)) were obtained from Aldrich Chemical Company. The molecular structures of DTAC, TX-100 and AOT-100 are shown in Scheme 1. Refined water was utilized for preparing test solutions for all measurements. The corrosion tests were performed on 1037 carbon steel. Rectangular specimens with dimensions 2.00 cm × 2.00 cm × 0.20 cm were utilized for weight loss estimations”. For electrochemical estimations, the exposed surface area of carbon steel was 1.00 cm^2^. Scheme 1 reports the structure, names and molecular formulas of the utilized surfactants.

**Fig. 1:**
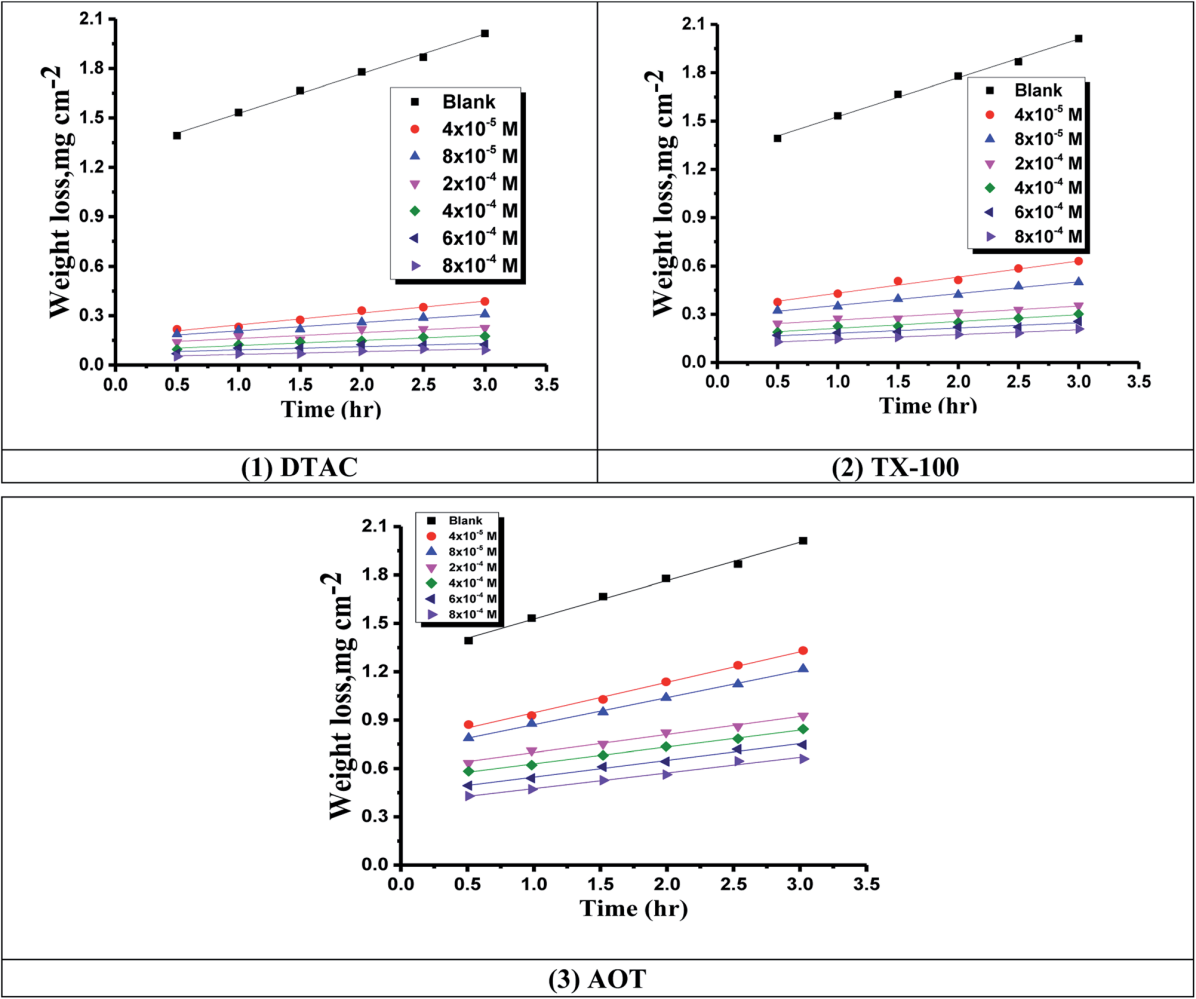
Time-WL bends of CS in half molar HCl in the existence and lack of various doses of (1) DTAC, (2) TX-100 and (3) AOT at 30 °C.

### 2.2 Methods

#### 2.2.1 Weight loss (WL) estimations

The WL tests were carried out in accordance with the American Society for Testing and Materials (ASTM) standard protocol. CS specimens were mechanically cleaned with 80 to 1200 grit sizes of emery papers and degreased with acetone, washed two times with distilled water and finally dried using filter papers. After precisely weighing, the specimens were dipped in 100 ml of 0.5 M HCl with and without altered concentrations of surfactants at 30 °C. After a distinctive submersion time period (30, 60, 90, 120, 150 and 180 min), the CS coins were taken out, washed with distilled water, dried and then weighted precisely. The tests were performed in triplicate and the average value of the WL was considered.

### 2.3. Electrochemical measurement

Three electrochemical techniques, “specifically potentiodynamic polarization (PDP), electrochemical impedance spectroscopy (EIS), and electrochemical frequency modulation (EFM), were utilized to study the corrosion behavior. All tests were conducted in an ordinary three cathodes glass cell. A Pt electrode as the counter electrode and a saturated calomel electrode as the reference electrode were utilized in this. The CS specimen was machined into a rectangular shape (1.00 cm × 1.00 cm × 0.30 cm) and fixed with epoxy resin taking off a working zone of 1.00 cm^2^, and the specimens were cleaned”, degreased, and washed as portrayed in WL estimations.

#### 2.3.1. PDP measurements

These were carried out using a Volta Lab “PGZ 100 system connected to a personal computer using the Volta Master 4 version 7.08 software for calculation. All the tests were performed at a temperature of 30 °C. The balance time leading to the consistent state of the specimens was 20 min and the open circuit potential (OCP) was noted”. The potentiodynamic bends were recorded from −900 to −200 mV at a scan rate 1 mV s^−1^.

#### 2.3.2. EIS and EFM measurements

These were carried out using a Gamry Instrument Series “G 750™ Potentiostat/Galvanostat/ZRA equipped with a Gamry framework system based on ESA400. Gamry applications include software EIS300 for EIS measurements, and EFM140 to calculate the corrosion current density and the Tafel constants for EFM measurements. A computer was utilized for collecting information. The Echem Analyst 5.5 software was utilized for plotting, graphing and fitting information. EIS estimations were carried out in the frequency range of 10^5^ to 10 Hz with an amplitude of 10 mV peak to peak using ac signals at a respective corrosion potential. EFM was carried out utilizing two frequencies 0.2 and 0.5 Hz”. The base frequency became 0.1 Hz, so the waveform repeats after 0.1 s. In this study, we make use of a perturbation sign with an amplitude of 10 mV for each perturbation frequency of 20 and 50 m Hz.

### 2.4 Quantum chemical calculation

HOMO energy “(highest occupied molecular orbital), LUMO energy (lowest unoccupied molecular orbital) and Fukui index estimations were performed using Materials Studio DMol^3^ version 4.4.0,^26,27^ a high-quality quantum mechanics computer program (available from Accelrys Inc., San Diego, CA). These estimations employed an *ab initio*, gradient-corrected functional (GGA) method with a double numeric plus polarization (DNP) basis set and a Becke One Parameter (BOP) functional. It is well known that the phenomena of electrochemical corrosion appear in an aqueous phase. For this reason, it is essential to incorporate the solvent effect in the computational calculations. In a comparable way, it is imperative to consider the impacts that can appear as much in the geometric properties as within the electrical ones. DMol^3^ incorporates certain COSMO”^28^ controls, which permit for the behavior of solvation impacts.

## 3. Results and discussion

### 3.1 Weight loss (WL) measurements

The WL values were used to calculate the corrosion rate (CR) in milli-meter consistent with year (mm per year) using the eqn (1):

where *K* = 8.75 × 10^4^.

The protection efficiency (% IE) and the degree of surface coverage (*θ*) were calculated using the following relation eqn (2) and (3):1
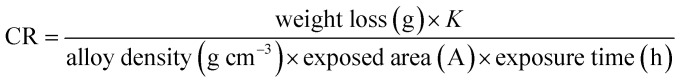
2
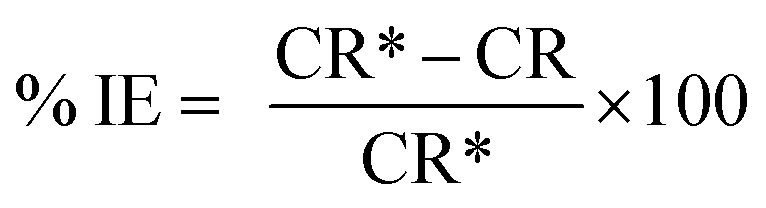
3
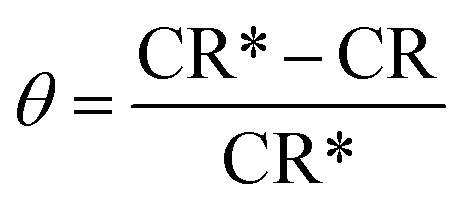
where CR* and CR are the corrosion rates of C-carbon steel with and without surfactants, respectively. Fig. 1 reports the WL-time bends for CS in 0.5 M HCl at various doses of the three surfactants at 30 °C.

Table 1 shows the dependence of “% IE on the altered concentrations of surfactants (AOT, TX-100 and DTAC) in the range from 4.0 × 10^−5^ mol l^−1^ to 8.0 × 10^−4^ mol l^−1^_._ From the data in this table, the following conclusions can be made”:

**Table 1:** WL for CS in 0.5 M HCl solution in the presence and absence of altered doses of surfactants at 30 °C

Compound	Conc., M	CR (mm per year)	*θ*	% IE
Blank	0.5	2.68 ± 0.0173	—	—
AOT	4.0 × 10^−5^	2.13 ± 0.0145	0.205	20.5
	8.0 × 10^−5^	1.94 ± 0.0145	0.276	27.6
	2.0 × 10^−4^	1.27 ± 0.0230	0.526	52.6
	4.0 × 10^−4^	1.17 ± 0.0155	0.563	56.3
	6.0 × 10^−4^	1.16 ± 0.0239	0.567	56.7
	8.0 × 10^−4^	1.08 ± 0.0153	0.597	59.7
TX-100	4.0 × 10^−5^	1.10 ± 0.0203	0.590	59.0
	8.0 × 10^−5^	0.82 ± 0.0014	0.694	69.4
	2.0 × 10^−4^	0.48 ± 0.0015	0.820	82.0
	4.0 × 10^−4^	0.46 ± 0.0023	0.828	82.8
	6.0 × 10^−4^	0.36 ± 0.0015	0.866	86.6
	8.0 × 10^−4^	0.341 ± 0.0023	0.873	87.3
DTAC	4.0 × 10^−5^	0.80 ± 0.0015	0.700	70.0
	8.0 × 10^−5^	0.56 ± 0.0014	0.791	79.1
	2.0 × 10^−4^	0.39 ± 0.0017	0.854	85.4
	4.0 × 10^−4^	0.35 ± 0.0015	0.869	86.9
	6.0 × 10^−4^	0.22 ± 0.0016	0.918	91.8
	8.0 × 10^−4^	0.19 ± 0.0012	0.929	92.9

(1) At a constant temperature, the % PE rises with improvement in the surfactant concentration.

(2) The lowest CR was obtained by DTAC and the highest by AOT-100; therefore, IE tends to decrease in the subsequent order: DTAC > TX-100 > AOT.

(3) The reticence action of the surfactants in “HCl can be simply considered as electrostatic adsorption^29^ and covalent bonding chemisorption. This action was attributed to the effect of chloride ions of DATC surfactants, chloride ions of acid solution and chemisorption of C_12_H_25_N^+^ (CH_3_)_3_, C_34_H_62_O_11_ and C_20_H_37_O_7_S^−^. In addition, other factors such as CMC and structure of surfactant might be affecting the inhibition activity”.

#### 3.1.1 Effect of temperature

**Fig. 2:**
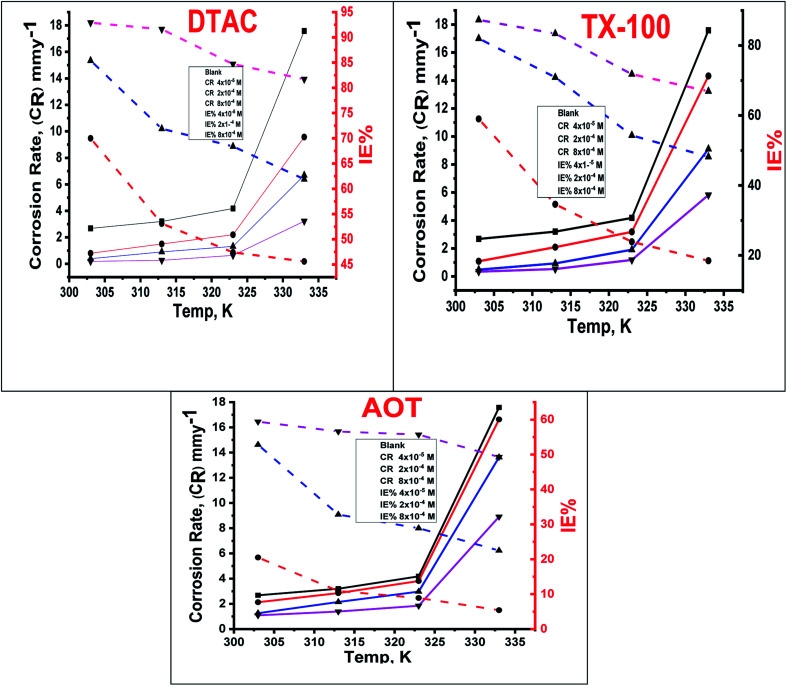
Variation of % IE and (CR) at different doses of utilized surfactants in 0.5 M HCl at different temperatures.

The effect of temperature, “in the range of 30–60 °C with an increment of 10 °C on both the corrosion rate and the % IE of different surfactants in 0.5 M HCl, was studied by WL measurements and was given in Fig. 2. In Fig. 2, we can see that increasing the temperature leads to an increase in the corrosion rate of CS both in free acid and inhibited acid solutions and a decrease in the % IE of surfactants, which suggested that corrosion inhibition of CS by the investigated surfactants is caused by the adsorption of surfactant molecules onto the CS surface, while at higher temperatures, the desorption of the investigated surfactants from the CS surface occurred”.^30^

#### 3.1.2 Kinetic parameters of the used surfactants on the CS surface

The inhibition properties of the used surfactants can be explained by means of kinetic model. The activation parameters were calculated from Arrhenius eqn (4) and transition state eqn (5):4
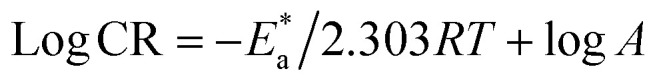
where *A* represents the pre-exponential factor.

**Fig. 3:**
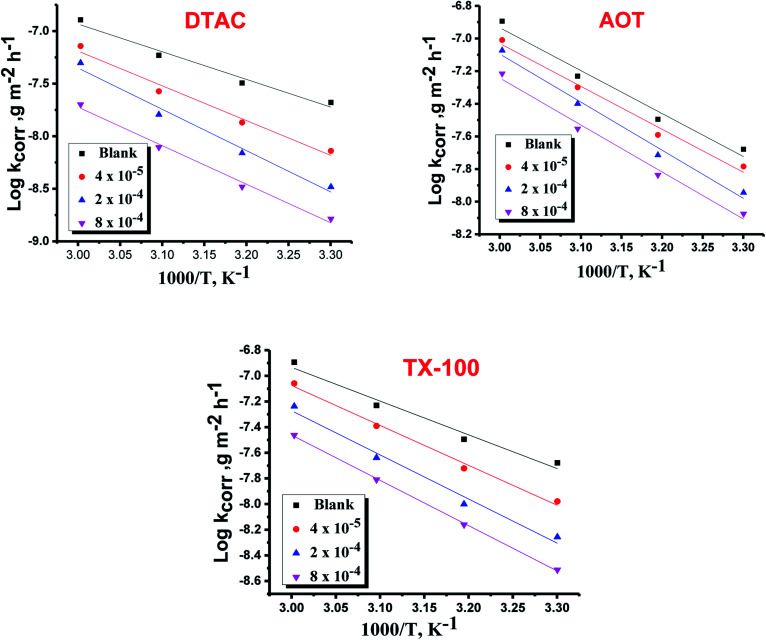
Arrhenius bends of variation of log *k*_corr._*vs.* 1/*T* for the dissolution of CS in half molar HCl in the presence and absence of different doses of DTAC, AOT and TX-100.

**Fig. 4:**
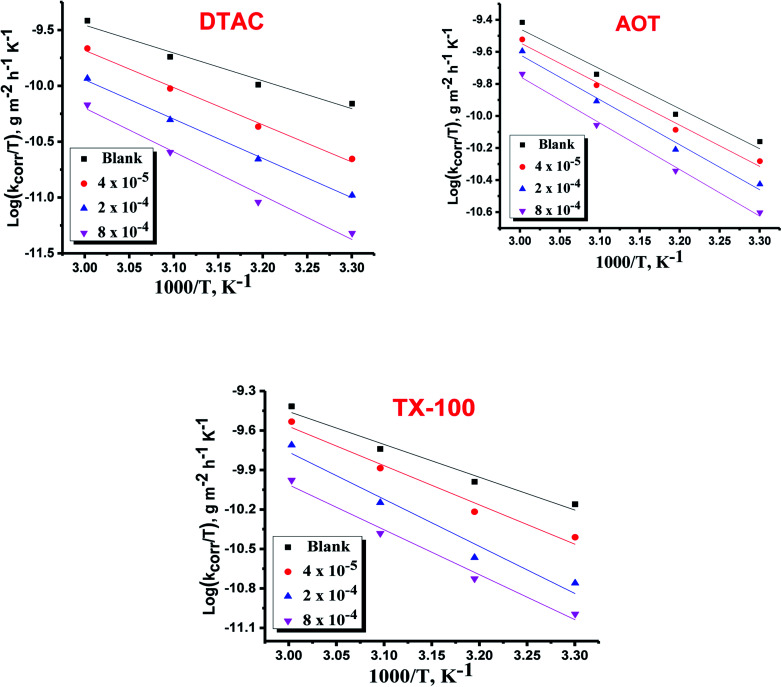
Variation of log *k*_corr._/*T vs.* 1/*T* for the dissolution of CS in 0.5 M HCl in the presence and absence of various doses of DTAC, AOT and TX-100.

5 CR = *RT*/*Nh* exp(ΔS*/*R*)exp(−ΔH*/*RT*)

where “*h* is Planck's constant, *N* is the Avogadro number, ΔS* is the entropy of activation, and ΔH* is the enthalpy of activation. The values of CR (in g m^−2^ h^−1^) were obtained at different temperatures, which is a linear function with 1/*T* and permits the calculation of the Arrhenius activation energy, 
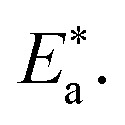
 The regression between log *k*_corr._ and 1/*T* was calculated, and Arrhenius plots of log *k*_corr._*vs.* 1/*T* for the blank and different concentrations of surfactants are shown in Fig. 3. All parameters are given in Table 2. The values of 
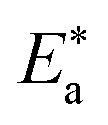
 in the presence of surfactants are higher than those in the uninhibited acid solution. The results agreed with that of previous study reported by Fouda *et al.*^31^ and Popova *et al*.^32^ for CS in 1 M HCl. The addition of surfactants to the solution increases the activation energy 
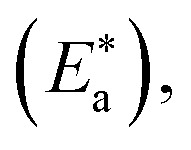
 as shown in Table 2 and the extent of the increase is proportional to the IE of the surfactant, indicating that the energy barrier for the corrosion reaction increases in the presence of these surfactants. This means that by addition of the surfactant in the acid solution, the corrosion reaction will be further pushed to surface sites that are characterized by higher values of activation energy 
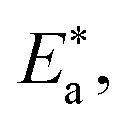
 indicating that CS corrosion occurs at the uncovered part of the surface. Thus, adsorption of the surfactant was assumed to occur on the higher energy sites,^33^ and the presence of surfactant, which results in the blocking of the active sites, must be associated with an increase in the activation energy, 
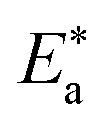
 which indicates the physical adsorption or weak chemical bonding between the surfactant molecules and the CS surface.^34^ The increase in 
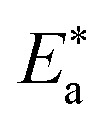
 in the presence of the surfactants indicates the physical adsorption that occurs in first stage,^35,36^ weak chemical bonding between the surfactants and the steel surface^37^ or due to the decrease in the adsorption of these surfactants with the increase in temperature.^38^ Generally, one can say that the nature and concentration of electrolytes greatly affect the activation energy for the corrosion process. A plot of log(*k*_corr._/*T*) *vs.* (1/*T*) gives a straight line with a slope of [−ΔH*/2.303*R*] and an intercept of [log(*R*/*Nh*) + ΔS*/2.303*R*]. The plots are shown in Fig. 4. The calculated values of ΔH* and ΔS* are given in Table 2. The values of ΔH* are positive indicating that the corrosion process is endothermic one. The values of 
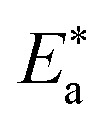
 are larger than the analogous values of ΔH*, indicating that the corrosion process must involve a gaseous reaction, simply the hydrogen evolution reaction, associated with a decrease in the total reaction volume”. These data verified the known thermodynamic relation among 
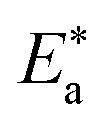
 and ΔH*^39^ which is as follows eqn (6):6
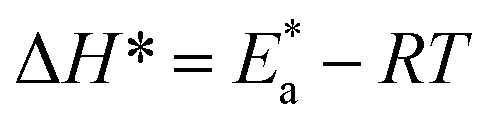


**Table 2:** Kinetic parameters for CS in 0.5 M HCl in the absence and presence of different doses of surfactants

Comp.	Conc., M	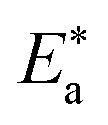 kJ mol^−1^	ΔH*, kJ mol^−1^	−ΔS*, J mol^−1^ K^−1^
Blank	0.5	50.4 ± 0.2309	47.7 ± 0.1453	236.3 ± 0.1453
AOT	4 × 10^−5^	50.4 ± 0.2028	49.3 ± 0.2333	233.3 ± 0.2729
	2 × 10^−4^	56.4 ± 0.20278	53.8 ± 0.2404	221.2 ± 0.1764
	8 × 10^−4^	56.8 ± 0.1732	54.2 ± 0.2028	218.4 ± 0.1528
TA-100	4 × 10^−5^	59.7 ± 0.2333	57.1 ± 0.2333	199.8 ± 0.1732
	2 × 10^−4^	71.2 ± 0.2603	68.5 ± 0.2309	178.2 ± 0.1764
	8 × 10^−4^	74.8 ± 0.2646	72.2 ± 0.2603	172.7 ± 0.1856
DTAC	4 × 10^−5^	63.3 ± 0.1528	60.7 ± 0.2333	202.4 ± 0.1764
	2 × 10^−4^	75.2 ± 0.2028	72.5 ± 0.1764	169.8 ± 0.1528
	8 × 10^−4^	78.2 ± 0.2048	76.2 ± 0.2404	159.5 ± 0.1453

The entropy of activation (ΔS*) is negative in each in the presence and absence of components, implying that the activated complex represented the rate determining step with respect to the association in place of the dissociation step. It means that a lower disorder took place whilst intending from reactants to the activated complex.^40^ In addition, the much less negative values of ΔS* in the presence of additives created a near-equilibrium corrosion system state.^41^

### 3.2 Electrochemical measurements

#### 3.2.1 PDP measurements

All electrochemical measurements were carried out using Volta Master 4, which calculates and displays *E*_corr._ (mV), *i*_corr._ (mA cm^−2^), *β*_a_ (mV), *β*_c_ (mV) and the corrosion rate (CR) in mm per year. This CR was calculated using the following relation eqn (7):^42^7
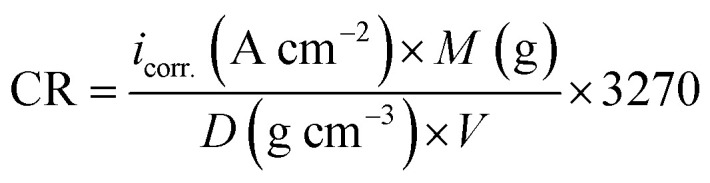
where “*i*_corr._ is the corrosion current density, *D* is the density of CS, *M* is the atomic mass of Fe and V is the valence entered in the Tafel dialogue box. With 3270 = 10 × [1 year (in seconds)/96497.8] and 96497.8 = 1 Faraday in Coulombs”. The % IE was calculated as follows eqn (8):8
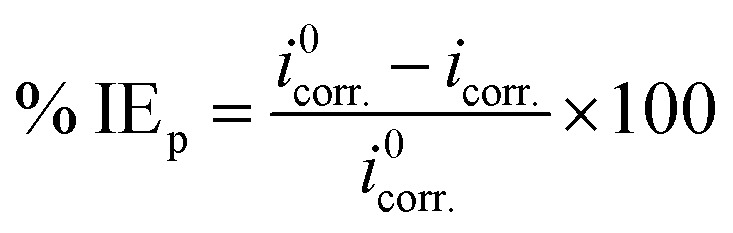
where *i*^0^_corr._ and *i*_corr._ are the corrosion current densities of uninhibited and inhibited solutions, respectively.

**Fig. 5:**
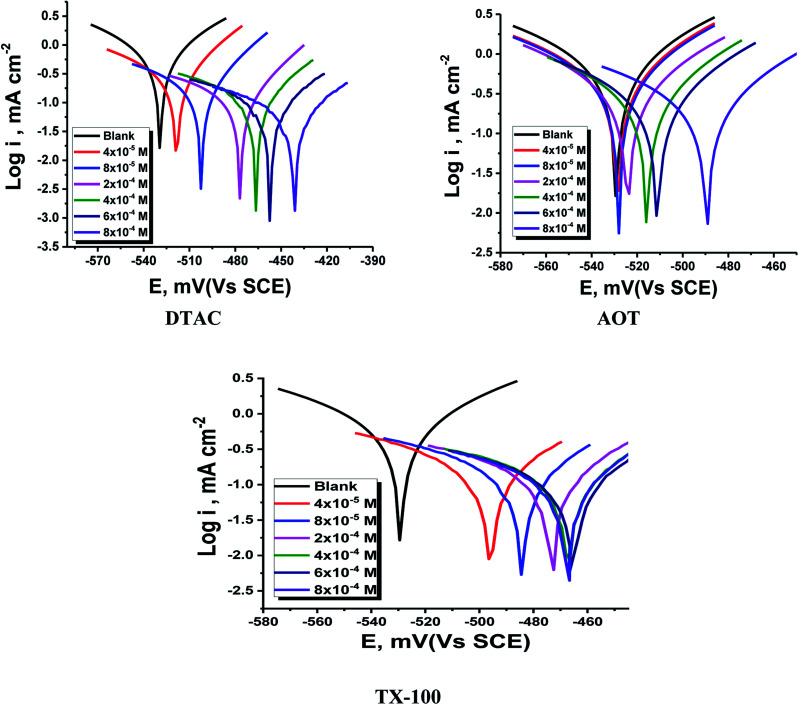
PDP for CS dissolution in half-molar HCl existence and absence of altered doses of DTAC, TX-100 and AOT at 30 °C.

The PDP curves for CS in 0.5 M HCl with and without “DTAC, TX-100 and AOT are shown in Fig. 5. It can be observed from these Figures that the presence of surfactants in 0.5 M HCl solution shifts the corrosion potential (*E*_corr._) to more positive values, while the polarization curves were shifted to lower current regions. These phenomena certify the inhibitory effect of these surfactants by increasing their concentrations in HCl and both cathodic and anodic reactions were inhibited by increasing the surfactant concentration. Moreover, the cathodic curves give approximately parallel lines, suggesting that the hydrogen discharge reaction slows down, its activation being controlled^43^ by surfactant addition in a HCl medium. The selected surfactants act as mixed-type inhibitors *i.e.*, promoting retardation of both anodic dissolution of CS and cathodic hydrogen discharge reactions. This was confirmed by the slow displacement of *E*_corr._ (less than 85 mV). By inspecting the data in Table 3, it can be observed that: (a) with the increase in surfactant concentration, *i*_corr._ values decrease gradually, (b) the anodic and cathodic Tafel slopes (*β*_c_ & *β*_a_)) are slightly changed after surfactant addition, and (c) this indicates that surfactants influence both anodic and cathodic processes;^44^ the IE increases reaching a maximum value of 86.46% at 8 × 10^−4^ M. The irregular trends of *β*_a_ and *β*_c_ values indicate the participation of more than one type of species adsorbed onto the metal surface”. The maximum inhibiting impact is achieved with the surfactant DTAC, and % IE values decrease as follows (Table 3): DTAC > TX-100 > AOT.

**Table 3:** PDP data of CS in half molar HCl and in the existence of different doses of surfactants at 30 °C

Comp.	Conc., M	−*E*_corr._, mV *vs.* SCE	*i*_corr._, μA cm^−2^	−*β*_c_, mV dec^−1^	*β*_a_, mV dec^−1^	*θ*	% IE	CR, μm per year
Blank	0.5	529.2 ± 0.1455	515.6 ± 0.1155	69.1 ± 0.2309	54.2 ± 0.1175	—	—	5976
AOT	4.0 × 10^−5^	528.72 ± 0.2333	405.4 ± 0.1732	72.4 ± 0.1453	51.3 ± 0.2028	0.2137	21.37	4641
	8.0 × 10^−5^	528.2 ± 0.1453	387.8 ± 0.2028	72.7 ± 0.1653	50.9 ± 0.3528	0.2479	24.79	4495
	2.0 × 10^−4^	516.6 ± 0.17634	283.9 ± 0.2603	68.1 ± 0.1553	52.6 ± 0.2028	0.4485	44.85	2427
	4.0 × 10^−4^	515.7 ± 0.1453	201.7 ± 0.1764	64.5 ± 0.1732	46.3 ± 0.1453	0.6088	60.88	2338
	6.0 × 10^−4^	511.1 ± 0.1732	190.9 ± 0.2028	67.9 ± 0.2309	48.6 ± 0.2906	06 298	62.98	2212
	8.0 × 10^−4^	489.5 ± 0.1732	181.5 ± 0.1732	67.9 ± 0.2028	50.3 ± 0.1732	0.6480	64.80	2103
TX-100	4.0 × 10^−5^	507.1 ± 0.1453	224.7 ± 0.2028	69.0 ± 0.2028	49.6 ± 0.2028	0.5642	56.42	2604
	8.0 × 10^−5^	496.7 ± 0.2333	179.4 ± 0.2309	71.3 ± 0.1732	51.9 ± 0.1732	0.6520	65.20	2051
	2.0 × 10^−4^	487.1 ± 0.1453	160.5 ± 0.1732	70.6 ± 0.2309	53.0 ± 0.2082	0.6887	68.87	1860
	4.0 × 10^−4^	484.5 ± 0.1000	140.0 ± 0.1453	72.1 ± 0.1453	49.5 ± 0.1732	0.7285	72.85	1622
	6.0 × 10^−4^	483.7 ± 0.1202	114.9 ± 0.1523	79.3 ± 0.2603	52.5 ± 0.2028	0.7772	77.72	1332
	8.0 × 10^−4^	479.3 ± 0.1732	101.7 ± 0.2603	81.5 ± 0.2309	49.6 ± 0.1764	0.8028	80.28	1179
DTAC	4.0 × 10^−5^	518.4 ± 0.1732	213.3 ± 0.1453	74.6 ± 0.2028	41.7 ± 0.2028	0.5863	58.63	2472
	8.0 × 10^−5^	502.3 ± 0.2309	130.9 ± 0.2333	77.5 ± 0.1202	38.6 ± 0.2128	0.7461	74.61	1517
	2.0 × 10^−4^	477.4 ± 0.2028	87.2 ± 0.1202	82.0 ± 0.1732	39.0 ± 0.2231	0.8309	83.09	1010
	4.0 × 10^−4^	471.5 ± 0.2028	85.1 ± 0.2309	78.5 ± 0.1752	45.9 ± 0.2124	0.8349	83.49	993
	6.0 × 10^−4^	452.2 ± 0.2028	82.3 ± 0.2028	89.2 ± 0.1522	45.1 ± 0.2227	0.8404	84.04	954
	8.0 × 10-4	445.5 ± 0.1732	69.8 ± 0.2028	80.4 ± 0.1202	45.4 ± 0.2325	0.8646	86.46	809

#### 3.2.2 EIS measurements

**Fig. 6:**
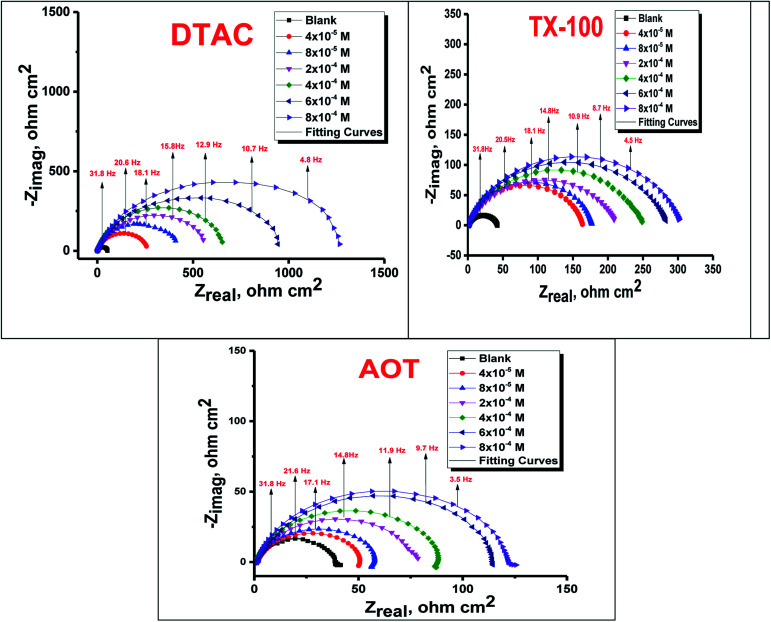
Nyquist bends for CS in half molar HCl in the absence and presence of DTAC, TX-100 and AOT surfactants at 30 °C.

**Fig. 7:**
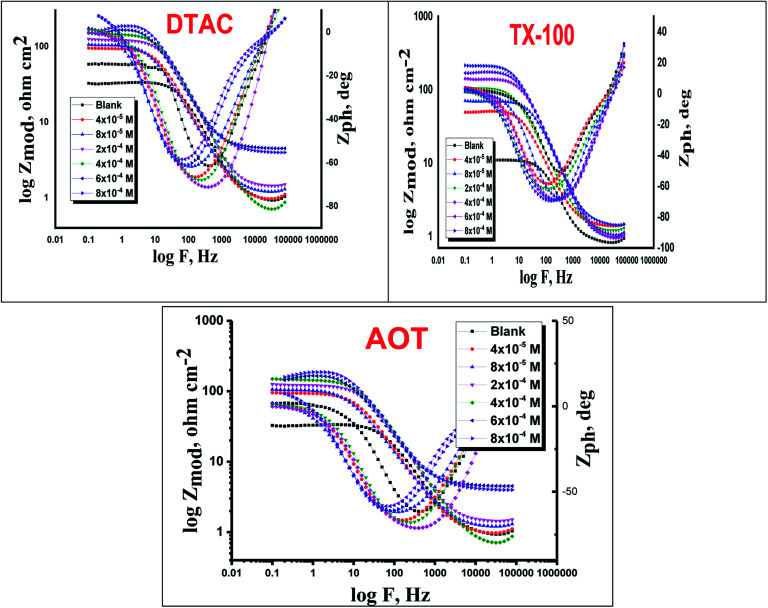
Bode bends for CS in half molar HCl in the absence and presence of DTAC, TX-100 and AOT at 30 °C.

**Fig. 8:**
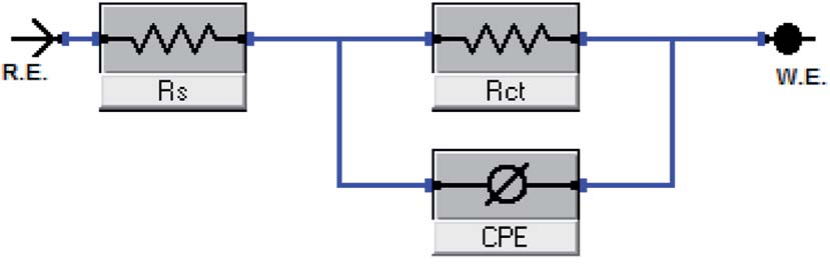
Equivalent circuit model utilized to fit EIS data.

The EIS provides protective layer stability, mechanistic and kinetic information for an electrochemical system under investigation. “The Nyquist impedance plots obtained for the CS electrode at respective corrosion potentials after 15 min immersion in 0.5 M HCl with and without altered concentrations of surfactants are shown in Fig. 6. These diagrams exhibit one capacitive loop appearing as a semicircle in Nyquist plots, being more pronounced with the increase in surfactant concentration. Bode plots given in Fig. 7 show that a single maximum of the phase angle corresponds to each capacitive loop in Nyquist plots. From Bode graphs plotted as phase *vs.* log *F*, it may observed that the phase angle does not exceed 90° (like for pure capacitive impedance).^45^ Consequently, log *Z vs.* log frequency Bode plots (Fig. 5) show two horizontal plateaus at the highest frequencies, which is attributed to log *R*_s_ (solution resistance), at the lowest frequencies that represent log(*R*_s_ + *R*_ct_). Furthermore, the Nyquist plots do not yield perfect semicircles as expected from the theory of EIS, the impedance loops measured are depressed semicircles with their centers below the real axis, where the kind of phenomenon is known as the “dispersing effect” as a result of frequency dispersion^46^ and mass transport resistance^47^ as well as electrode surface heterogeneity, resulting from surface roughness, impurities, dislocations, grain boundaries, adsorption of surfactants, formation of porous layers,^48–51^*etc.*, so one constant phase element (CPE) is substituted for the capacitive element, to explain the depression of the capacitance semi-circle, to give a more accurate fit. Impedance data are analyzed using the circuit in Fig. 8, in which *R*_s_ represents the electrolyte resistance, *R*_ct_ represents the charge-transfer resistance of the constant phase element (CPE). According to Hsu and Mansfeld,^52^ the correction of capacity to its real data is as follows eqn (9):

9 *C*_dl_ = *Y*_o_ (*ω*_max_)^*n*−1^

where “*Y*_o_ is the CPE coefficient, *ω*_max_ is the frequency at which the imaginary part of impedance (−*Z*_i_) has a maximum^53^ and *n* is the CPE exponent (phase shift). The data obtained from fitted spectra are listed in Table 4”. The value of (*θ*) was calculated from the EIS data using the following relation eqn (10):10
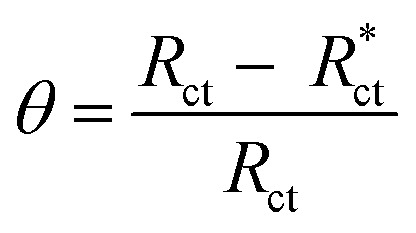


**Table 4:** EIS data of CS in half molar HCl in the absence and presence of different doses of surfactants at 30 °C

Comp.	Conc., M	*R*_S_, Ω cm^2^	*Y*_o_, μΩ^−1^ s^*n*^ cm^−2^	*n*	*R*_ct_, Ω cm^2^	*C*_dl_, μF cm^−2^	*θ*	% IE
Blank	0.5	1.946 ± 0.0173	542	0.865	32.1 ± 0.1453	437.0 ± 0.2333	—	—
AOT	4.0 × 10^−5^	2.165 ± 0.0145	381	0.867	47.09 ± 0.1764	236.5 ± 0.1453	0.318	31.8
	8.0 × 10^−5^	2.184 ± 0.0233	331	0.872	56.43 ± 0.2309	202.6 ± 0.1732	0.432	43.2
	2.0 × 10^−4^	1.814 ± 0.0145	259	0.876	74.11 ± 0.1732	183.1 ± 0.1732	0.567	56.7
	4.0 × 10^−4^	2.344 ± 0.0230	165	0.890	84.91 ± 0.2028	120.6 ± 0.1562	0.622	62.2
	6.0 × 10^−4^	3.284 ± 0.0155	137	0.893	110 ± 0.2128	99.2 ± 0.1322	0.708	70.8
	8.0 × 10^−4^	3.103 ± 0.0239	129	0.895	119.9 ± 0.1732	93.3 ± 0.1202	0.732	73.2
TX-100	4.0 × 10^−5^	1.102 ± 0.0153	133	0.856	175.0 ± 0.1453	93.1 ± 0.1732	0.817	81.7
	8.0 × 10^−5^	1.757 ± 0.0203	78	0.895	190.2 ± 0.1623	58.9 ± 0.1202	0.831	83.1
	2.0 × 10^−4^	1.835 ± 0.0155	73	0.897	217.9 ± 0.1403	55.52 ± 0.2309	0.853	85.3
	4.0 × 10^−4^	2.433 ± 0.0145	63	0.898	268.2 ± 0.2028	42.5 ± 0.1553	0.88	88
	6.0 × 10^−4^	2.527 ± 0.0172	59	0.929	303.2 ± 0.2309	38.9 ± 0.1651	0.894	89.4
	8.0 × 10^−4^	2.706 ± 0.0161	56	0.946	316.8 ± 0.1523	38.7 ± 0.2028	0.899	89.9
DTAC	4.0 × 10^−5^	1.711 ± 0.0155	113	0.834	187.5 ± 0.1851	79.4 ± 0.1553	0.0829	82.9
	8.0 × 10^−5^	1.835 ± 0.0203	73	0.839	382.0 ± 0.1233	48.9 ± 0.1732	0.916	91.6
	2.0 × 10^−4^	1.894 ± 0.0153	57	0.840	535.3 ± 0.1553	40.2 ± 0.2309	0.94	94
	4.0 × 10^−4^	1.926 ± 0.0239	55	0.844	637.0 ± 0.1163	37.2 ± 0.2028	0.95	95
	6.0 × 10^−4^	2.426 ± 0.0155	53	0.857	867.5 ± 0.2028	36.7 ± 0.1453	0.963	96.3
	8.0 × 10^−4^	2.673 ± 0.0153	52	0.869	1135 ± 0.2309	36.6 ± 0.1553	0.972	97.2

The % IE was calculated as follows eqn (11):11
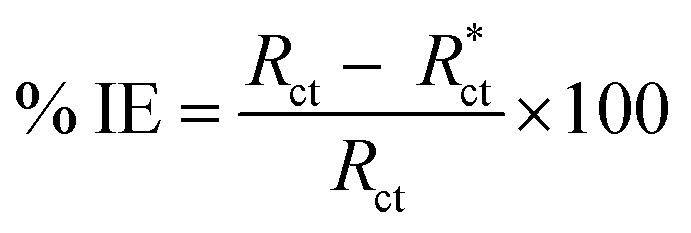
where *R*_ct_ and 
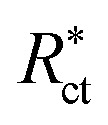
 are the resistance in the presence and absence of surfactants, respectively.

The data given in Table 4 demonstrate that the *R*_s_ data are slightly associated with the *R*_ct_ values. “By increasing the surfactant concentrations, the *R*_ct_ values increase and the calculated *C*_dl_ values decrease, which causes an increase in *θ* and IE. The high *R*_ct_ values are generally associated with a slower corroding system.^54^ The decrease in *C*_dl_ suggests that the surfactant molecule function by adsorption at the metal/solution interface and can result in a decrease in the local dielectric constant and/or an increase in the thickness of the electrical double layer.^55^ It was also observed from the Table that the (*n*) value varies directly with the surfactant concentration, while the reverse is the case with *Y*_o_, the (*n*) value being a measure of the roughness of the surface of the working electrode resulting from the adsorption of the surfactant molecules”.^56^

#### 3.2.3 EFM measurements

**Fig. 9:**
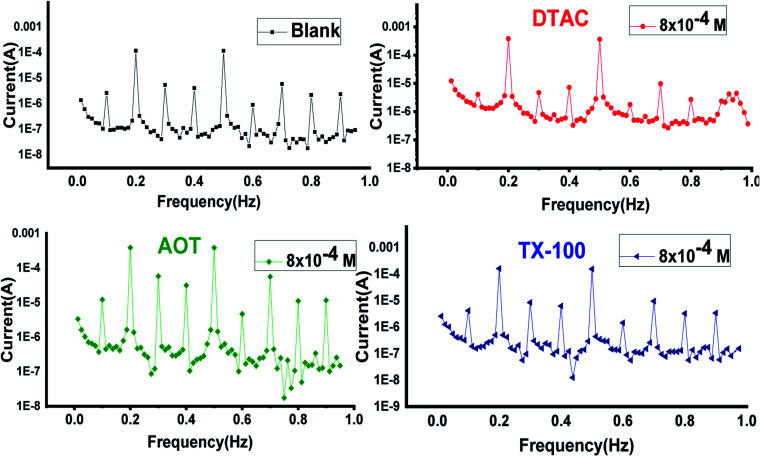
EFM bends for CS in half molar HCl in the absence and presence of 8 × 10^−4^ M dose of DTAC, AOT and TX-100 at 30 °C.

EFM is an electrochemical technique in which two sinusoidal potential signals are summed and applied to a corrosion sample *via* a Potentiostat. “The great strength of the EFM is the causality factor which serves as an internal check on the validity of the EFM measurement.^57^ With the causality factors, the experimental EFM data can be verified. EFM is a nondestructive corrosion measurement like EIS; it is a small signal ac technique. Unlike EIS, however, two sine waves (at different frequencies) are applied to the cell simultaneously. The results of EFM experiments are a spectrum of current response as a function of frequency. The spectrum is called the intermodulation spectrum. The spectra contain current responses assigned for harmonica and intermodulation current peaks. The larger peaks were used to calculate the corrosion current density (*i*_corr._), the Tafel slopes (*β*_c_ and *β*_a_) and the causality factors (CF-2 and CF-3). Intermodulation spectra obtained from EFM measurements are presented in Fig. 9 for 0.5 M HCl in the absence and presence of 8 × 10^−4^ M of AOT, TX-100 and DTAC, respectively. Similar curves were obtained for other concentrations of surfactants (not shown). As can be seen from Table 5, the corrosion current densities decrease by increasing the concentrations of the studied surfactants. The % IE_EFM_ calculated from eqn (8) increase by increasing the studied surfactant concentrations. The causality factors in Table 5 are very close to theoretical values, which according to the EFM theory^58^ should guarantee the validity of Tafel slopes and corrosion current densities. Fig. 9 shows the % IE recorded for DTAC, TX-100 and AOT at a concentration of 8 × 10^−4^ M using the four different techniques, namely, WL, PP, EIS and EFM”. The calculated % IE found is in excellent agreement.

**Table 5:** EFM parameters obtained for CS in the presence and absence of different doses of surfactants in 0.5 M HCl at 30 °C

Comp.	Conc., M	*i*_corr._, μA cm^−2^	*β*_c_, mV dec^−1^	*β*_a_, mV dec^−1^	CF-2	CF-3	*θ*	% IE	CR , μm per year
Blank	0.00	488.9 ± 0.2028	126 ± 0.1453	106 ± 0.2906	1.86	2.95	—	—	5763
AOT	4 × 10^−5^	394.3 ± 0.1553	96 ± 0.1732	88 ± 0.2027	1.67	2.98	0.193	19.3	4575
	8 × 10^−5^	321.9 ± 0.1653	107 ± 0.2309	88 ± 0.1763	1.84	2.92	0.342	34.2	3735
	2 × 10^−4^	272.8 ± 0.1453	102 ± 0.2309	87 ± 0.2028	1.80	3.21	0.4420	44.2	3165
	4 × 10^−4^	238.6 ± 0.2431	92 ± 0.1732	86 ± 0.2082	1.33	2.82	0.512	51.2	2768
	6 × 10^−4^	208.7 ± 0.2055	97 ± 0.2102	88 ± 0.2028	1.74	2.73	0.573	57.3	2422
	8 × 10^−4^	117.4 ± 0.1452	95 ± 0.1732	83 ± 0.2123	1.87	2.87	0.759	75.9	1362
TX-100	4 × 10^−5^	88.3 ± 0.1742	98 ± 0.2101	93 ± 0.2234	1.44	2.77	0.819	81.9	1025
	8 × 10^−5^	87.1 ± 0.2102	95 ± 0.2423	89 ± 0.2131	1.46	3.01	0.822	82.2	1011
	2 × 10^−4^	83.1 ± 0.2209	97 ± 0.2512	91 ± 0.2028	1.56	2.77	0.830	83	965
	4 × 10^−4^	70.0 ± 0.2010	107 ± 0.1202	93 ± 0.1732	1.72	2.64	0.857	85.7	812
	6 × 10^−4^	56.2 ± 0.1453	98 ± 0.2333	85 ± 0.2309	1.86	3.32	0.885	88.5	652
	8 × 10^−4^	50.88 ± 0.1353	85 ± 0.1453	73 ± 0.1453	1.99	5.684	0.896	89.6	590
DTAC	4 × 10^−5^	79.1 ± 0.1725	114 ± 0.2027	105 ± 0.2028	1.67	3.01	0.838	83.8	691
	8 × 10^−5^	42.4 ± 0.2333	114 ± 0.2022	106 ± 0.1453	1.55	3.13	0.913	91.3	493
	2 × 10^−4^	41.3 ± 0.2121	132 ± 0.1027	113 ± 0.1732	1.91	2.69	0.916	91.6	476
	4 × 10^−4^	30.7 ± 0.2028	130 ± 0.1121	115 ± 0.1453	2.01	2.77	0.937	93.7	357
	6 × 10^−4^	28.6 ± 0.2228	118 ± 0.1327	113 ± 0.2028	1.63	2.67	0.941	94.1	333
	8 × 10^−4^	27.8 ± 0.2108	149 ± 0.1125	134 ± 0.2027	1.91	2.62	0.943	94.3	323

**Fig. 10:**
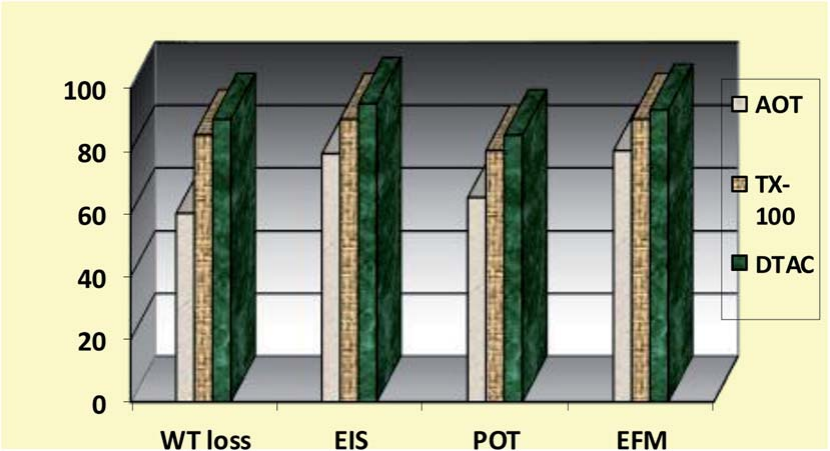
Comparison of “% IE (recorded using EFM, EIS, PP and WL measurements) obtained for the three surfactants during CS corrosion in 0.5 M HCl solutions containing 8 × 10^−4^ M of these three surfactants at 30 °C”, respectively.

The order of the % IE found for the *R*_ct_ data is DTAC > TX-100 > AOT (Fig. 10). “The % IE, calculated from EIS results, show the same trend as those obtained from PP measurements. The difference in % IE from two methods may be attributed to the different surface status of the electrode in two measurements. EIS was performed at the rest potential, while in polarization measurements, the electrode potential was polarized to high overpotential and non-uniform current distributions, resulting from cell geometry, solution conductivity, counter and reference electrode placement, *etc.*, which will lead to the difference between the electrode area undergoing polarization and the total area”.^59^

### 3.3 Adsorption of surfactants

Adsorption of surfactants on solid surfaces can regulate their hydrophobicity, surface charge, and different key properties such as corrosion protection that govern the interfacial process. “In general, adsorption is governed by several forces such as covalent bonding, electrostatic attraction, hydrogen bonding or non-polar interactions between the adsorbed species, lateral associative interaction, solvation, and desolvation.^60^ The total adsorption is usually the cumulative result of some or all the above-mentioned forces.^61^ The standard free energy of adsorption can be written as follows” eqn (12):^62^12
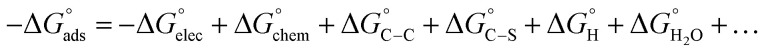
where “
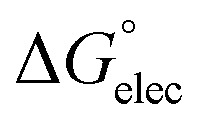
 is the electrostatic interaction term, 
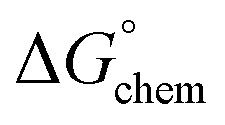
 the chemical term due to covalent bonding, 
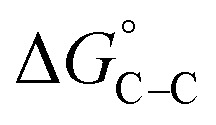
 the free energy gained upon association of methyl groups in the hydrocarbon chain, 
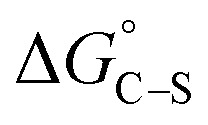
 the free energy due to interactions between the hydrocarbon chains and hydrophobic sites on the solid, 
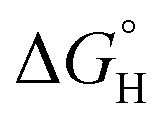
 the hydrogen bonding term and 
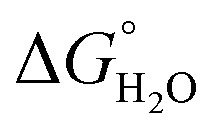
 the term owing to dissolution or solvation of the adsorbate species, or any species displaced from the interface due to adsorption”. In the following, the major forces involved in surfactant adsorption are discussed:

#### 3.3.1 Driving forces for surfactant adsorption

For each surfactant–solid system, several of the above-mentioned terms can be operative depending on the solid and the surfactant type, surfactant concentration, electrolyte, pH, temperature, *etc.*:

(i) Electrostatic interactions 
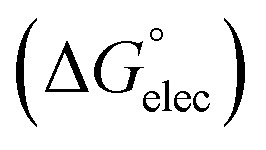
: in systems where the ionic surfactants and the solid particles are charged, electrostatic interactions play a governing role in the adsorption process.

(ii) Chemical interactions 
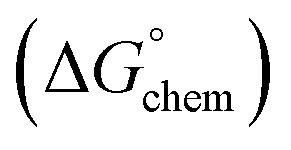
: chemical interaction is another important driving force for adsorption of surfactants on the solid particles. Compared to other driving forces, this interaction is specific to certain systems where covalent bonding can occur between the surfactant and the solid.

(iii) Hydrophobic lateral interactions 
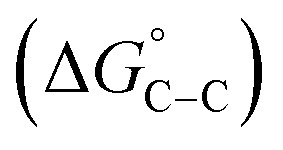
: “At a concentration above a threshold value, analogous to the aggregation in the bulk, surfactant molecules tend to form two dimensional aggregates at the solid/liquid interface, causing an abrupt increase in the adsorption density. These aggregates have been called “hemi-micelles”^63^ or, in general, “colloids” for surface colloids.^64^ The driving force for adsorption 
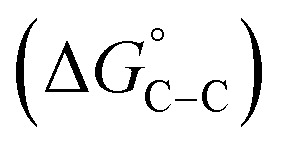
 results from the free energy of transferring the hydrocarbon chains from the aqueous environment into the hydrophobic interior of the aggregates. 
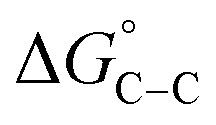
 can be represented as a linear function of the energy gained per –CH_2_ group”.^65^

(iv) Hydrophobic interaction between the hydrocarbon chains and hydrophobic sites on the solid 
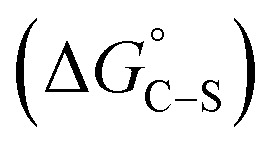
: “the hydrophobic interaction 
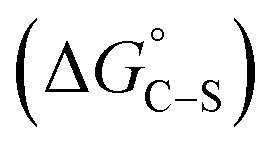
 between the alkyl chain of a surfactant and the hydrophobic sites on the solid becomes a significant factor for surfactant adsorption on fully or partially hydrophobic surfaces. In this case, the surfactant molecules attach to the hydrophobic sites with the hydrocarbon chains aligned parallel to the surface at low concentrations and normal to the surface at higher concentrations. Such an adsorption process often results in a two-step isotherm”.

(v) Hydrogen bonding 
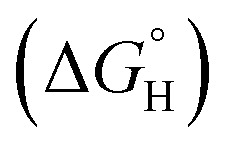
: “Hydrogen bonding between surfactant species and the solid surface species could occur in systems containing hydroxyl, phenolic, carboxylic and amine groups on the surfactant. For instance, adsorption of a nonionic surfactant such as ethoxylated alcohol and sugar-based alkyl glucoside on oxides has been proposed to involve hydrogen bonding.^66,67^ It should be noted that for adsorption due to hydrogen bonding to take place, the bond formed between the surfactant functional groups and mineral surfaces should be stronger than that formed between the mineral and interfacial water molecules”.

(vi) Desolvation energy 
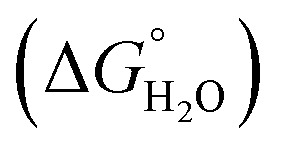
: “When a hydrated head group of the surfactant transfers from the bulk to the mineral–solution interfacial region, partial removal of water molecules from the secondary solvation shell around the surfactant head groups can occur. In contrast to other driving forces, desolvation energy due to such a process is unfavorable for the adsorption process”.

### 3.4 Mechanism of adsorption^68^

The adsorption of ionic surfactants on oppositely charged surface could be taking the following path: (i) “At low surfactant concentrations, the adsorption is due to electrostatic interaction between individual isolated charged monomeric species and the oppositely charged solid surface. (ii) Surfactant species begin to form surface aggregates, colloids (surface colloids), including hemi-micelles and ad-micelles, due to lateral interactions between hydrocarbon chains. Thus, the additional driving force resulting from the lateral association with the electrostatic interaction is still active. (iii) When the solid surface is electrically neutralized by the adsorbed surfactant ions, the electrostatic attraction is no longer operative, and adsorption takes place due to lateral attraction alone with a reduced slope. (iv) When the surfactant concentration reaches critical micelle concentration, the surfactant monomer activity becomes constant and any further increase in the concentration contributes only to the micellization in solutions and it does not change the adsorption density. The adsorption in this region is mainly through lateral hydrophobic interaction between the hydrocarbon chains. In steps (iii) and (iv), surfactant molecules adsorb with a reversed orientation (head groups facing the bulk solution), resulting in a decrease in the hydrophobicity of the particles in this region. The pH plays a very significant role in controlling the adsorption of ionic surfactants. Thus, the adsorption of anionic surfactants is higher on positively charged surfaces (pH below isoelectric point (IEP)) than on negatively charged surfaces, while the cationic surfactants adsorb more on negatively charged surfaces.^69,70^ The molecular structure of surfactants does influence its adsorption behavior markedly. Most nonionic surfactants contain polar groups that form hydrogen bonds with the hydroxyl groups on the solid surface. Since the hydrogen bonding is weaker than the electrostatic interaction, the adsorption of the nonionic surfactant to most solids is less than that of ionic surfactants. Nonionic surfactants exhibit adsorption like those of cationic surfactants, except for a sharp increase in step III of the adsorption mechanism because of the absence of electrostatic interactions. The adsorption of the nonionic surfactants also depends on the pH and number of the hydrophilic groups and the hydrocarbon chain length”. Several adsorption isotherms have been assessed, and the Langmuir adsorption isotherm become determined to be the excellent description of the adsorption conduct of the applied surfactants, which obey the subsequent eqn (13):

13 *C*_inh/*θ*_ =1/*K*_ads_ + *C*_inh_

**Fig. 11:**
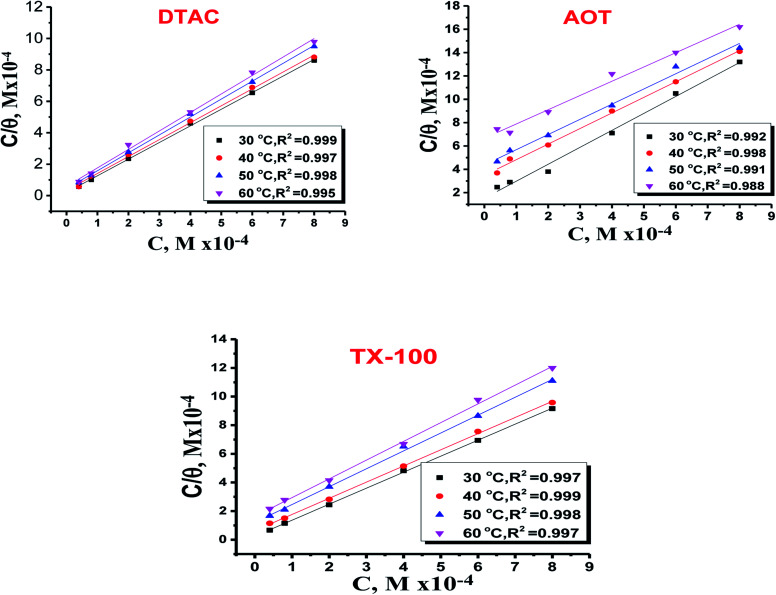
Langmuir bends for the adsorption of DTAC, AOT and TX-100 on CS in half molar HCl at different temperatures.

14
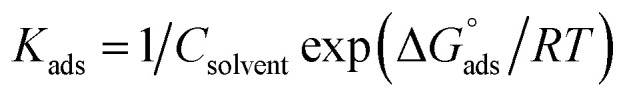
where “*C*_inh_ is the surfactant concentration, *θ* is the fraction of the surface coverage, *K*_ads_ is the modified adsorption equilibrium constant, which can be related to the free energy of adsorption 
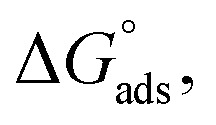
 and *C*_solvent_ is the molar concentration of solvent, which in the case of water is 55.5 mol l^−1^. The 
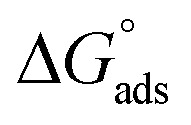
 value was calculated according to eqn (14). Fig. 11 shows the dependence of the fraction of the surface coverage (*C*/*θ*) as a function of the concentration (*C*) of AOT, TX-100 and DTAC. The degrees of surface coverage (*θ*) were evaluated from EIS measurements using eqn (10) and are given in Table 6. The 
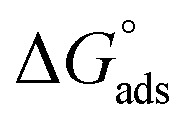
 values are negative, suggesting the spontaneity of the adsorption process. The regression coefficient is *R*^2^ = 0.999. It is well known that values of 
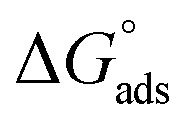
 in the order of 20 kJ mol^−1^ or lower indicate physisorption, while those in the order of 40 kJ mol^−1^ or higher involve charge sharing or charge transfer from the surfactant molecules to the metal surface to form a coordinate type of bond.^71,72^ The calculated values of 
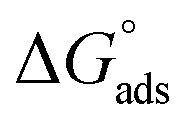
 for DTAC are around −38.1 to 37.3 kJ mol^−1^, for TX-100 approximately −36.8 to 35.2 kJ mol^−1^ and for AOT-100 approximately −32.2 to 31.4 kJ mol^−1^, so physical and chemical adsorption (mixed type) may proposed.

**Table 6:** Parameters for the adsorption of surfactants in half molar HCl on the CS at different temperatures

Surfactant	Temp.	*K*_ads_ ×10^−4^ M^−1^	Slope	*R* ^2^	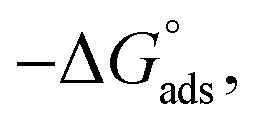 kJ mol^−1^	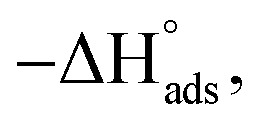 kJ mol^−1^	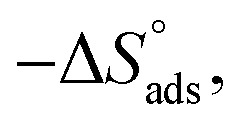 *J* mol^−1^ K^−1^
AOT	303	0.65	1.3	0.991	32.2 ± 0.1732	37.77 ± 0.2082	38.68 ± 0.2333
	313	0.28	1.3	0.993	31.1 ± 0.2028		
	323	0.23	1.2	0.998	31.6 ± 0.1453		
	333	0.15	1.2	0.976	31.4 ± 0.2028		
TX-100	303	4.03	1.1	0.999	36.8 ± 0.1028	52.15 ± 0.1732	70.82 ± 0.1453
	313	1.57	1.1	0.999	35.6 ± 0.1732		
	323	0.83	1.2	0.998	35.0 ± 0.2028		
	333	0.60	1.3	0.998	35.2 ± 0.1453		
DTAC	303	4.88	1.0	0.999	37.3 ± 0.1732	27.47 ± 0.2333	12.10 ± 0.2028
	313	1.93	1.0	0.994	36.5 ± 0.2028		
	323	1.39	1.0	0.995	37.4 ± 0.1732		
	333	1.08	1.1	0.998	38.1 ± 0.1001		

### 3.5 Surface analyses results

**Fig. 12:**
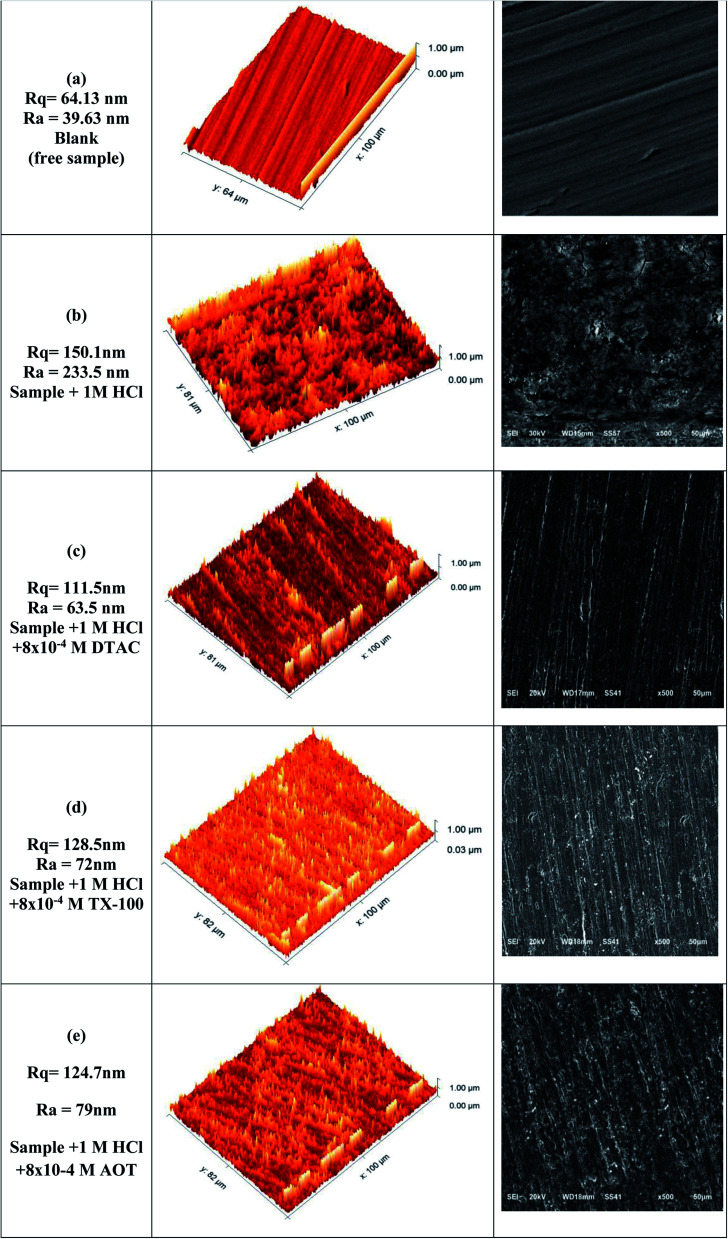
(a–e) SEM and AFM images for blank (free sample), sample + 1 M HCl, sample + 1 M HCl + 8 × 10^−4^ DTAC, sample + 1 M HCl + 8 × 10^−4^ TX-100, sample + 1 M HCl + 8 × 10^−4^ AOT.

Surface analysis was conducted by both electron scanning microscopy (SEM) and atomic electron microscopy (AFM), as depicted in Fig. 12. “SEM examination was used to acquire a thorough knowledge of the corrosion morphology prior to and after immersion in 1 M HCl solution with and without DTAC, TX-100, and AOT at 30 °C. Fig. 12(a) shows a polished CS surface with no fractures or pits save for a few polished abrasions. Fig. 12(b) depicts the CS specimen following immersion in 1 M HCl without surfactants, revealing a badly degraded CS surface with a larger number of fractures and pits. Fig. 12(c–e) shows a CS specimen following immersion in 1 M HCl with DTAC, TX-100, and AOT, which showed a substantial improvement with a lower number of cracks and pits as compared to the CS surface without surfactants, due to the inhibitive layer development on the CS surface. The fact that the DTAC-inhibited surface is smoother than the TX-100 and AOT-inhibited surfaces indicates that DTAC is a superior inhibitor than TX-100 and AOT. Fig. 12(a–e) shows 3D micrographs of the CS surface obtained from AFM with and without surfactants. Fig. 12(a) depicts a polished CS surface with an average roughness of 39.63 nm. The CS surface is shown in Fig. 12(b) after being immersed in a 1 M HCl solution without DTAC, TX-100, or AOT; the average roughness is 233.5 nm. Fig. 12(c–e) show the CS surface after submersion in 1 M HCl solution containing DTAC, TX-100, and AOT, with average roughness values of 63.5, 72.0, and 79.0 nm, respectively”. DTAC has a lower average roughness than that of TX-100, whereas AOT has better adsorption than that of DTAC.

### 3.6 Computational study and corrosion mechanism

Lower values of ionization potential “*I*_P_ (−*E*_HOMO_) are likely to indicate a tendency of the molecule to donate electrons to appropriate acceptor molecules with low energy or empty electron orbital. The higher the values of electron affinity *E*_A_ (−*E*_LUMO_), the stronger the electron accepting abilities of the molecules. However, the hydrophobic properties of the long hydrocarbon tail could be associated with the formation of a protective film that drastically reduces the corrosion process. Pearson introduced the quantities of electronic hardness (*η*) and softness (*σ*) in his hard–soft-acid–base principle^73^ (HSAB) in the early stage of the reactivity theory. The species are classified as soft (hard) if their valence electrons are easy (hard) to polarize or to remove and the relationship between hardness or softness and the chemical reactivity was given by the HSAB principle. A soft base will interact favorably with a soft acid, sharing electrons, to form bonds of covalent character. Hard acids prefer hard bases and form bonds dominated by electrostatic forces, or ionic character. The concepts of electronegativity (*χ*) and global hardness (*η*)”^74^ are given as follows eqn (15) and (16):15
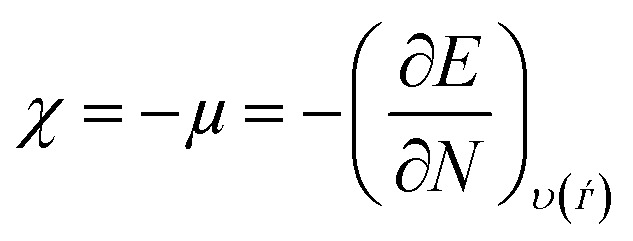
16
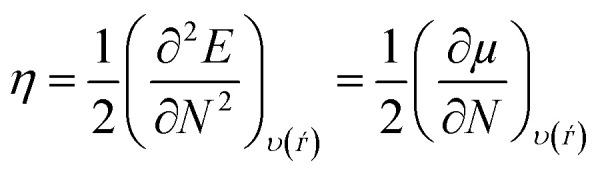
where “*μ* is the chemical potential, *E* is the total energy, *N* is the number of electrons, and *ν*(*ѓ*) is the external potential of the system. The global hardness (*η*), softness (*σ*), and chemical potential (*μ*) were calculated in terms of *I*_P_ and *E*_A_”^75^ as follows eqn (17) and (18):17
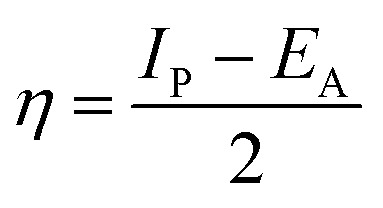
18
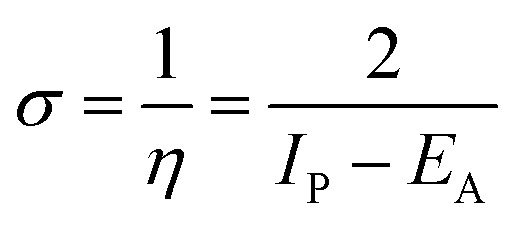
19
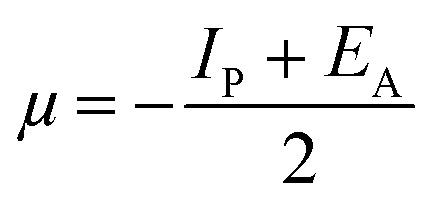


**Fig. 13:**
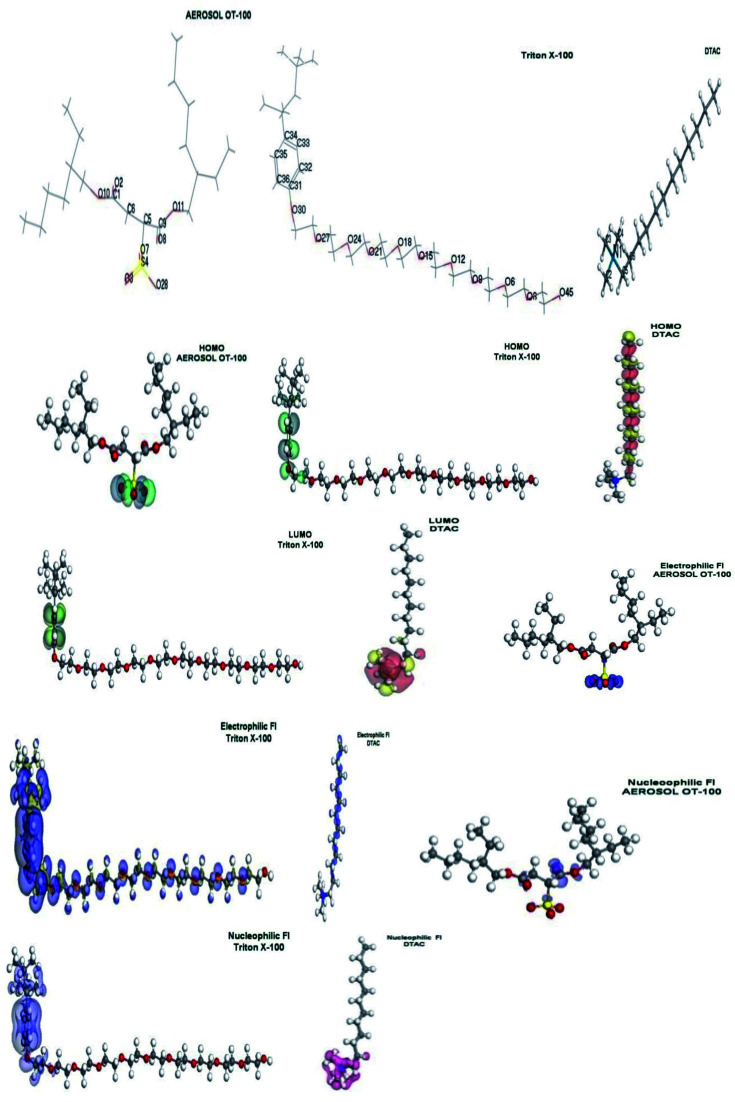
Molecular orbital plots as well as the active sites for electrophilic and nucleophilic attack for AOT, TX-100 and DTAC, respectively.

In Table 7, certain quantum-chemical parameters related to the molecular electronic structure are presented, “which were obtained by means of the application of POP/DNP basis set in each one of the interest systems_._ The results for the above-mentioned calculations in a gaseous phase as well as in a liquid phase are presented. These results show the effect of applying a calculation in the liquid phase. From these results, TX-100 exhibits the lowest value of global hardness (high softness) in liquid phases compared with the cationic part of DTAC and the anionic part of AOT. This suggested a higher tendency of covalent adsorption of TX-100 to occur than other used surfactants. It means that this one has a higher reactivity. The role of the counter ions on the adsorption of ionic surfactants is an important factor. High hardness of Cl^−^ (counter ion effect) and cationic part of DTAC suggested higher tendency of electrostatic adsorption of DTAC to occur (cooperative effect) than other used surfactants that lead to a high % IE. The feasible adsorption of organic cations in the presence of the halide ions is due to the formation of intermediate bridge, the negative ends of the halide metal dipoles being oriented towards the solution. The dipoles of the surface compound formed are oriented with their negative ends towards the solution, whereby setting up an additional potential difference between the metal and the solution. This will shift the zero-charge potential positively. This shift will make the charge on the metal surface more negative and facilitates the adsorption of positively charged quaternary ammonium compound [C_12_H_25_N^+^(CH_3_)_3_] by the formation of ionic bonds. Cl^−^ ions act as adsorption mediators for bonding the two positive partners, the metal surface and the positively charged ammonium compound. This gives rise to the formation of an adsorption composite film, in which the chloride ions (Cl^−^) are sandwiched between the metal and positively charged part of the surfactant.^76^ This film acts as a barrier facing the corrosion process, as shown in Fig. 13. Anionic part of AOT is a softer base than Cl^−^ thus it attached with metals (softer) *via* covalent adsorption. The anionic part of AOT suggested a competitive effect with Cl^−^ ions of the HCl solution. In this case (showing the anodic process of iron corrosion), Cl^−^ ion-catalyzed iron dissolution occurs”. The anionic part of AOT can retard the corrosion process by adsorption onto a positively charged steel surface.

**Table 7:** Quantum-chemical descriptors for surfactants found with DFT meted

Quantum-chemical descriptors										
			*I*_P_, eV	*E*_A_, eV	Δ*E*, eV	*η*, eV	*μ*, eV	*σ*, eV	*X*, eV	*−E*_tot_.
AOT	Anionic part	Gas phase	0.55	2.848	3.403	1.70	0.588	1.15	−0.58	1709.2
		Liquid phase	5.342	0.982	4.36	2.18	0.459	−3.162	3.162	1709.3
	Na^+^ counter	Gas phase	36.23	6.99	29.24	9.62	0.104	−21.61	*21.61*	162.19
		Liquid phase	29.81	0.756	29.05	14.53	0.069	−15.28	*15.28*	162.2
TX-100	Cationic part	Gas phase	5.276	0.838	4.438	2.219	0.451	−3.057	3.057	2160.1
		Liquid phase	4.74	0.41	4.33	3.748	0.461	−2.58	2.58	2160.0
DTAC		Gas phase	8.158	2.934	5.224	2.612	0.383	−5.546	5.546	646.3
		Liquid phase	6.700	0.795	7.495	2.17	0.267	−2.952	2.952	646.4
	Cl^−^ counter	Gas phase	−2.33	19.08	16.75	8.375	0.119	10.705	−5.35	460.23
		Liquid phase	4.53	12.22	16.75	8.375	0.119	3.85	−3.85	460.35

The anodic process of iron corrosion eqn (20)–(22):

20 Fe + Cl^−^ ⇄ FeCl_ads_^−^

21 FeCl_ads_^−^ → Fe Cl^+^ + 2e

22 Fe Cl^+^ ⇄ Fe^2+^ + Cl^−^

From Fig. 13, we can observe that:

The HOMO location in the cationic part of DTAC surfactants is mostly distributed on the hydrophobic part. “The LUMO location in the cationic part of DTAC is mostly distributed on the nitrogen cation that the preferred sites for the nucleophilic attack through metallic negative centers. The position of the surfactant (lying vertically) could be the reason for the high surfactant efficiency. The HOMO location in the nonionic surfactant is mostly distributed on the benzene ring (hydrophobic part) and oxygen atom attached to it. The LUMO location in the nonionic surfactant is mostly distributed on the benzene ring (hydrophobic part). However, the molecular structure of TX-100 suggests that they are able to adsorb onto the metal surface through two lone pair of electrons of the polar hydrophilic head groups by forming hydrogen bonds. Oxygen atoms can form a protective layer (which blocs the active sites of corrosion on the metal surface). The position of the surfactant (lying flat) could be the reason for the high surfactant efficiency. The HOMO location in anionic surfactants is mostly distributed on oxygen atoms attached to the sulfur atom, indicating that the preferred sites for the electrophilic attack through metallic positive center are located on these oxygen atoms. The LUMO location in the anionic surfactant is mostly distributed on oxygen atoms of the nearest acetate group to the sulfur atom, indicating that the preferred sites for the nucleophilic attack through metallic negative centers are located on the oxygen atoms. From the above, it is mentioned that a hydrophilic metal surface attracts a large hydrophilic head group of chosen surfactants. Table 7 shows that the values of Δ*E* dropped in the following order: AOT^+^ > TX-100 > DTAC, showing that DTAC inhibits more effectively than TX-100 and AOT. From this table, because DTAC has a greater *σ* and lower *η* value, it can be inferred that this molecule has a higher potential to be adsorbed on steel surfaces. It should also be noticed that DTAC has the maximum number of electrons, indicating that this chemical has a strong theoretical capacity to suppress steel corrosion. These findings indicate that the active centers on the steel surface are extremely well adsorbent of this chemical. Among the theoretical models proposed to compute local reactivity indices are Fukui functions that make it possible to rationalize the reactivity of individual molecular orbital contributions, thus to account for the response of the whole molecular spectrum and not only of the frontier orbitals. Frontier orbital electron densities on atoms provide a useful means for the detailed characterization of donor–acceptor interactions. In the case of a donor molecule, *f*^−^(*r*) electrophilic electron density corresponds to reactivity with respect to electrophilic attack or when the molecule loss electrons and in the case of an acceptor molecule, *f*^+^(*r*) nucleophilic electron density corresponds to the reactivity with respect to nucleophilic attack. However, frontier electron densities can strictly be used only to describe the reactivity of different atoms in the same molecule. The highest FI values are presented in Table 8. The most susceptible sites for electrophilic attack are located on C10, C11, C12 and C13 atoms in case of DTAC, oxygen atoms and carbon atoms of benzene ring in case of TX-100 and O3, O7, O28, and S4 in case of AOT”. In addition, susceptible sites are experiential to be confronted by anions or nucleophilic attack, positioned on C2, C3, C4, C5 and N1 of DTAC, C32, C33, C35, and C36 in case of TX-100 and O10 and O12 in case of AOT (Fig. 11).

**Table 8:** The highest FI values

“The highest Fukui indices values for the three surfactants by Hirshfeld methods in liquid phase calculated with BOP/DNP basis set”								
AOT			TX-100			DTAC		
	Liquid phase			Liquid phase			Liquid phase	
	*f* ^*−*^(*r*)	*f* ^*+*^(*r*)		*f* ^*−*^(*r*)	*f* ^*+*^(*r*)		*f* ^*−*^(*r*)	*f* ^*+*^(*r*)
C1	0.003	0.048	O3	0.010	−0.001	N1	0.000	0.034
O2	0.016	0.054	O6	0.010	−0.001	C2	0.001	0.077
O3	0.226	0.050	O9	0.011	−0.001	C3	0.000	0.054
S4	0.105	0.039	O12	0.011	−0.001	C4	0.001	0.062
C5	0.020	−0.026	O15	0.011	−0.001	C5	0.007	0.059
C6	0.007	−0.039	O18	0.011	−0.001	C6	0.012	0.018
O7	0.245	0.026	O21	0.011	−0.001	C7	0.020	0.009
O8	0.027	0.019	O24	0.012	0.000	C8	0.032	0.005
C9	0.006	0.054	O27	0.018	0.004	C9	0.041	0.002
O10	0.007	0.179	O30	0.091	0.031	C10	0.052	0.001
O11	0.011	0.162	C31	0.070	0.065	C11	0.054	0.000
C12	0.004	0.024	C32	0.063	0.116	C12	0.057	0.000
O28	0.244	0.038	C33	0.051	0.107	C13	0.051	0.000
			C34	0.077	0.061	C14	0.046	0.000
			C35	0.051	0.117	C15	0.035	0.000
			C36	0.054	0.111	C16	0.029	0.000
			O45	0.002	0.002			

### 3.7 Monte Carlo (MC) simulation

**Fig. 14:**
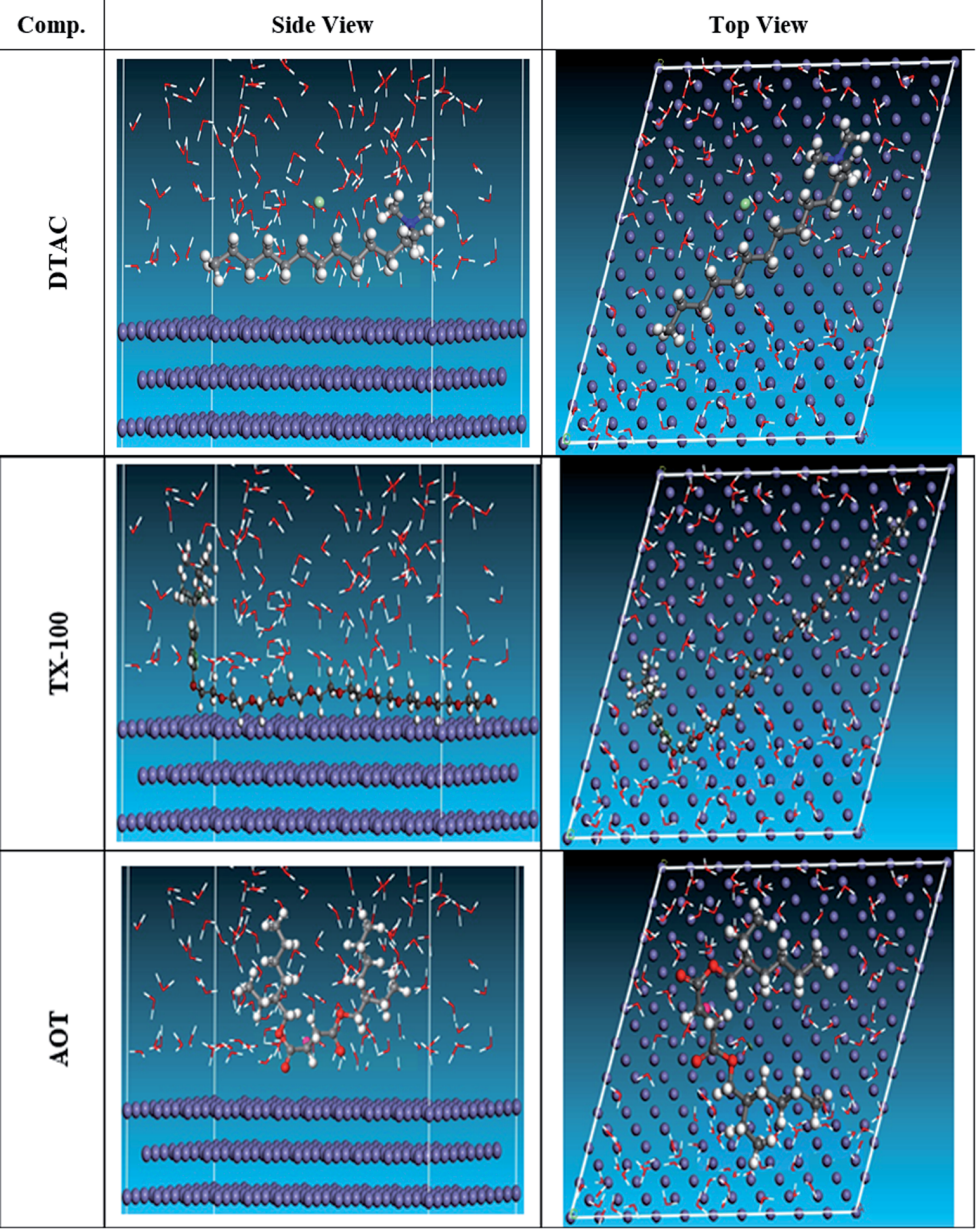
The most appropriate configuration for the adsorption of the surfactant molecules on the Fe (110) substrate received by an adsorption locator module.

The MC simulation was performed to study the adsorption behavior of the three inhibitors on the Fe (110) surface. Fig. 14 shows the most suitable nonionic surfactant configurations simulated in water solution adsorbed on the Fe (110) substrate attained by an adsorption locator module. According to the equilibrium configurations of the three inhibitors adsorbed onto the Fe (110) surface, we can draw a conclusion that surfactants can be absorbed onto the Fe surface through their oxygen atoms. In this way, the covering with surfactants, consequently preventing the surface from contact with water, can reduce the exposure of the Fe surface. Therefore, the corrosion inhibition is achieved by this factor. It can be inferred that the inhibitor molecules will form a water-proof film on the Fe surface after addition to the solution. The Adsorption Locator Study Table 9 output contain the following columns: structure: the configurations of the adsorbate components on the Fe surface. Total energy: the total energy, in kcal mol^−1^, of the Fe surface–non ionic surfactant–water configuration. The Fe surface energy is taken as zero. The total energy is defined as the sum of the energies of the adsorbate components, the rigid adsorption energy, and the deformation energy. Adsorption energy: the energy, in kcal mol^−1^, released (or required) when the relaxed adsorbate components are adsorbed onto the Fe surface. This is the sum of the rigid adsorption energy and the deformation energy for the adsorbate components. Rigid adsorption energy: the energy, in kcal mol^−1^, released (or required) when the unrelaxed adsorbate components (before the geometry optimization step) are adsorbed onto the Fe surface. Deformation energy: the energy, in kcal mol^−1^, released when the adsorbed components are relaxed on the Fe surface. d*E*_ad_/d*N*_i_: the energy, in kcal mol^−1^, of Fe surface-adsorbate configurations where one of the adsorbate components has been removed. Table 9 shows that the DTAC compound has the maximum binding energy, followed by TX-100, while AOT was the least effective compound in the simulation experiment. The inhibitory trend is still changing, with the adsorption energy increasing in the order of DTAC > TX-100 > AOT. Hence, the better stability formed complicated happened with DTAC and accordingly will increase its inhibition efficiency and that is steady with experimental results. In addition, the simulation was conducted in the presence of water species, with the same compound inhibition order. This demonstrates that DTAC inhibits most of the other chemicals in all of the systems studied. The results given in Table 9 reflect that the average value of adsorption energies is higher of order DTAC-water > AOT-water > TX-100-water system. In addition, the average energy needs to release DTAC are higher than those of other surfactants.

**Table 9:** Outputs and descriptors estimated by the Monte Carlo simulation for the adsorption of surfactants on iron (110)

Surf.,	Total energy	Adsorption energy	Rigid adsorption energy	Deformation energy	Compound d*E*_ad_/d*N*_i_	water d*E*_ad_/d*N*_i_
DTAC	−2057.9	−2195.2	−2127.18	−67.825	−84.8	−11.271
TX-100	−1260.0	−1370.0	−1330.00	−40.00	−20.00	−9.70
AOT	−1260.0	−1375.0	−1330.00	−45.38	−32.50	−8.25

## 4. Conclusions

DTAC, AOT and TX-100 showed good performance as corrosion inhibitors in hydrochloric acid media. The inhibition is because of the adsorption of the surfactant molecules onto the CS surface and blocking off of its active sites. Adsorption of the surfactants suits the Langmuir isotherm model. The results obtained from WL, PDP, EIS, and EFM tests are in reasonably excellent agreement and display improved surfactant performance with the growing surfactant concentration. PDP records display that the applied surfactants act as mixed-kind inhibitors in half molar HCl. The theoretical examination of molecules in each gaseous section and liquid section indicated the difference among AOT, TX-100, and DTAC, consistent with the HSAB principle. SEM combined with EDX and AFM analyses of the morphology of the CS surface revealed the presence of a persistent and insoluble adherent layer that prevents the electrolyte from reaching the metal's surface. Monte Carlo simulations reveal a strong interaction between the surfactants studied and the CS surface.

## Author contributions

A. S. Fouda, designated the experiments, carried out the experiments and data analysis, wrote the manuscript, discussion and edits the manuscript.

## Conflicts of interest

The author declares no competing interests.

## Supplementary Material

## References

[cit1] Oddo J. E., Tomson M. B. (1982). J. Pet. Technol..

[cit2] Ridd B. , Blakset T. J., Queen D., Corrosion, NACE*,* (1998). p. 78

[cit3] Banerjee G., Malhotra S. N. (1992). Corrosion.

[cit4] El Azhar M., Mernari B., Traisnel M., Bentiss F., Lagrenee M. (2001). Corros. Sci..

[cit5] Olivares O., Likhanova N. V., Gomez B., Navarrete J., Llanos-Serrano E. A., Hallen J. M. (2006). Appl. Surf. Sci..

[cit6] Trasatti S. (1992). Electrochim. Acta.

[cit7] Popova A., Sokolova E., Raicheva S., Christov M. (2003). Corros. Sci..

[cit8] Lagrenee M., Mernari B., Bouanis M., Traisnel M., Bentiss F. (2002). Corros. Sci..

[cit9] Tamil Selvi S., Raman V., Rajendran N. (2003). J. Appl. Electrochem..

[cit10] Kissi M., Bouklah M., Hammouti B., Benkaddour M. (2006). Appl. Surf. Sci..

[cit11] Kraka E., Cremer D. (2000). J. Am. Chem. Soc..

[cit12] Karelson M., Lobanov V. (1996). Chem. Rev..

[cit13] Hinchliffe A. , Chemical Modelling from Atoms to Liquids, John Wiley & Sons, New York, 1999

[cit14] Dabosi F., Derbali Y., Etman M., Srhiri A., Savignac A. de, J. (1991). Appl. Electrochem..

[cit15] El Achouri M., Hajji M. S., Kertit S., Essassi M., Salem M., Coudert R. (1995). Corros. Sci..

[cit16] Vasudevan T., Muralidharan S., Alwarappan S., Iyer S. V. K. (1995). Corros. Sci..

[cit17] El Achouri M., Hajji M. S., Salem M., Kertit S., Aride J., Coudert R., Essassi E. M. (1996). Corrosion.

[cit18] Migahed M. A., Al-Sabagh A. M. (2009). Chem. Eng. Commun..

[cit19] Free M. L. (2002). Corrosion.

[cit20] Saleh M., Atia A. A. (2006). J. Appl. Electrochem..

[cit21] Wang W. L., Free M. L. (2004). Corros. Sci..

[cit22] Atia A. A., Saleh M. M. (2003). J. Appl. Electrochem..

[cit23] Soror T. Y., El-Ziady M. A. (2002). Mater. Chem. Phys..

[cit24] Li X., Tang. L., lie H., Mu G., Lie G. (2008). Mater. Lett..

[cit25] Delley B. (1990). J. Chem. Phys..

[cit26] Delley B. (2000). J. Chem. Phys..

[cit27] Mulliken R. S. (1995). J. Chem. Phys..

[cit28] Ma H., Chen S., Yin B., Zhao S., Liu X. (2003). Corros. Sci..

[cit29] Volt Master 4 Manual, 2000

[cit30] Cao C. N. (2004). Chem. Ind. Eng..

[cit31] Fouda A. S., El-Gharkawy E. S., Ramadan H., El-Hossiany A. (2021). Biointerface Res. Appl. Chem..

[cit32] Popova A., Sokolova E., Raicheva S., Christov M. (2003). Corros. Sci..

[cit33] Fouda A. S., Abdel-Latif E., Helal H. M., El-Hossiany A. (2021). Russ. J. Electrochem..

[cit34] Li X. H., Mu G. N. (2005). Appl. Surf. Sci..

[cit35] Fouda A. S., Ibrahim H., Rashwaan S., El-Hossiany A., Ahmed R. M. (2018). Int. J. Electrochem. Sci..

[cit36] Larabi L., Benali O., Harek Y. (2007). Mater. Lett..

[cit37] Fouda A. S., Eissa M., El-Hossiany A. (2018). Int. J. Electrochem. Sci..

[cit38] Riggs O. L., Hurd R. M. (1967). Corrosion.

[cit39] Fouda A. S., Ahmed R. E., El-Hossiany A. (2021). Prot. Met. Phys. Chem. Surf..

[cit40] Musa A. Y., Kadhum A. A. H., Mohamad A. B., daud A. R., Takriff M. S., Kamarudin S. K. (2009). Corros. Sci..

[cit41] Khaled M. A., Ismail M. A., El-Hossiany A. A., Fouda A. S. (2021). RSC Adv..

[cit42] Fouda A. S., Abd El-Maksoud S. A., El-Hossiany A., Ibrahim A. (2019). Int. J. Electrochem. Sci..

[cit43] Fouda A. S., El-Ghaffar M. A. A., Sherif M. H., El-Habab A. T., El-Hossiany A. (2020). Prot. Met. Phys. Chem. Surf..

[cit44] Khaled K. F. (2003). Electrochim. Acta.

[cit45] Fouda A. S., El-Maksoud S. A., El-Hossiany A., Ibrahim A. (2019). Int. J. Electrochem. Sci..

[cit46] Growcock F. B., Jasinski J. H. (1989). J. Electrochem. Soc..

[cit47] Fouda A. S., Abdel Azeem M., Mohamed S. A., El-Hossiany A., El-Desouky E. (2019). Int. J. Electrochem. Sci..

[cit48] Mehaute A. H., Grepy G. (1983). Solid State Ionics.

[cit49] Motawea M. M., El-Hossiany A., Fouda A. S. (2019). Int. J. Electrochem. Sci..

[cit50] Hsu C. H., Mansfeld F. (2001). Corrosion.

[cit51] Fouda A. S., El-Dossoki F. I., El-Hossiany A., Sello E. A. (2020). Surf. Eng. Appl. Electrochem..

[cit52] Gamry Echem Analyst Manual, 2003

[cit53] Bosch R. W., Hubrecht J., Bogaerts W. F., Syrett B. C. (2001). Corrosion.

[cit54] Fouda A. S., Abd El-Maksoud S. A., Belal A. M., El-Hossiany A., Ibrahium A. (2018). Int. J. Electrochem. Sci..

[cit55] Somasundaran P., Grieves R. B. (1975). AIChE Symp. Ser..

[cit56] Fouda A. S., El-Wakeel A. M., Shalabi K., El-Hossiany A. (2015). Elixir Corros. Day.

[cit57] Fuerstenau D. W. , The chemistry of bio surfaces, Marcel Dekker, New York, 1971. p. 143

[cit58] Fuerstenau D. W., Healy T. W., Somasundaran P. (1964). Trans. AIME.

[cit59] Zerga B., Attayibat A., Sfaira M., Taleb M., Hammouti B., Ebn Touhami M., Radi S., Rais Z. (2010). J. Appl. Electrochem..

[cit60] Somasundaran P., Kunjappu J. T. (1989). Colloids Surf..

[cit61] Lin I. J., Somasundaran P. (1971). J. Colloid Interface Sci..

[cit62] Zhang L., Somasundaran P., Mielczarski J., Mielczarski E. (2002). J. Colloid Interface Sci..

[cit63] James R. O., Healy T. W. (1972). J. Colloid Interface Sci..

[cit64] Zhang R., Somasundaran P. (2006). Adv. Colloid Interface Sci..

[cit65] Koopal L. K., Lee E. M., Böhmer M. R. (1995). J. Colloid Interface Sci..

[cit66] Fan A.-X., Somasundaran P., Turro N. J. (1997). Langmuir.

[cit67] Donahue F. M., Nobe K. (1965). J. Electrochem. Soc..

[cit68] Kamis E., Belluci F., Latanision R. M., El-Ashry E. S. H. (1991). Corrosion.

[cit69] Fouda A. S., Rashwan S., El-Hossiany A., El-Morsy F. E. (2019). J. Chem., Biol. Phys. Sci..

[cit70] Ló pez D. A., Simison S. N., de Sánchez S. R. (2005). Corros. Sci..

[cit71] Shalabi K., Abdel-Galil E., El-Askalany A. H., Abdallah Y. M. (2022). J. Mol. Liq..

[cit72] Elgyar O. A., Ouf A. M., El-Hossiany A., Fouda A. S. (2021). Biointerface Res. Appl. Chem..

[cit73] Parr R. G., Pearson R. G. (1983). J. Am. Chem. Soc..

[cit74] Pearson R. G. (1988). Inorg. Chem..

[cit75] Sastri V. S., Perumareddi J. R. (1997). Corros. Sci..

[cit76] Soror T. Y., El-Ziady M. A. (2002). Mater. Chem. Phys..

